# The chemotherapeutic drug methotrexate selects for antibiotic resistance

**DOI:** 10.1016/j.ebiom.2021.103742

**Published:** 2021-12-11

**Authors:** Jónína S. Guðmundsdóttir, Elizabeth G.A. Fredheim, Catharina I.M. Koumans, Joachim Hegstad, Po-Cheng Tang, Dan I. Andersson, Ørjan Samuelsen, Pål J. Johnsen

**Affiliations:** a Department of Pharmacy, Faculty of Health Sciences, UiT The Arctic University of Norway, Tromsø, Norway; b Institute of Biology, Leiden University, Leiden, Netherlands; c Research and Development Division, Department of Microbiology and Infection Control, University Hospital of North Norway, Tromsø, Norway; d Department of Medical Biochemistry and Microbiology, Uppsala University, Uppsala, Sweden; e Norwegian National Advisory Unit on Detection of Antimicrobial Resistance, Department of Microbiology and Infection Control, University Hospital of North Norway, Tromsø, Norway

**Keywords:** Chemotherapeutic drugs, *E. coli*, Antibiotic resistance, Methotrexate, Resistance evolution

## Abstract

**Background:**

Understanding drivers of antibiotic resistance evolution is fundamental for designing optimal treatment strategies and interventions to reduce the spread of antibiotic resistance. Various cytotoxic drugs used in cancer chemotherapy have antibacterial properties, but how bacterial populations are affected by these selective pressures is unknown. Here we test the hypothesis that the widely used cytotoxic drug methotrexate affects the evolution and selection of antibiotic resistance.

**Methods:**

First, we determined methotrexate susceptibility (IC_90_) and selective abilities in a collection of *Escherichia coli* and *Klebsiella pneumoniae* strains with and without pre-existing trimethoprim resistance determinants. We constructed fluorescently labelled pairs of *E. coli* MG1655 differing only in trimethoprim resistance determinants and determined the minimum selective concentrations of methotrexate using flow-cytometry. We further used an experimental evolution approach to investigate the effects of methotrexate on *de novo* trimethoprim resistance evolution.

**Findings:**

We show that methotrexate can select for acquired trimethoprim resistance determinants located on the chromosome or a plasmid. Additionally, methotrexate co-selects for genetically linked resistance determinants when present together with trimethoprim resistance on a multi-drug resistance plasmid. These selective effects occur at concentrations 40- to >320-fold below the methotrexate minimal inhibitory concentration.

**Interpretation:**

Our results strongly suggest a selective role of methotrexate for virtually any antibiotic resistance determinant when present together with trimethoprim resistance on a multi-drug resistance plasmid. The presented results may have significant implications for patient groups strongly depending on effective antibiotic treatment.

**Funding:**

PJJ was supported by 10.13039/100007465UiT The Arctic University of Norway and the Northern Norway Regional Health Authority (SFP1292–16/HNF1586–21) and JPI-EC-AMR (Project 271,176/H10). DIA was supported by the 10.13039/501100004359Swedish Research Council (grant 2017–01,527). The publication charges for this article have been funded by a grant from the publication fund of UiT The Arctic University of Norway.

**Research in context**

**Evidence before this study**

We searched PubMed and Web of Science for the following search terms: Methotrexate + trimethoprim + resistan*; Cytostatic* + resistan* + antibiotic*; Cytostatic* + cross + resistan* + antibiotic; Chemotherapy + driving + resistan* + antibiotic*; drivers/driving + antimicro* + resistan*; fecal + cancer + trimethoprim in May 2020. We searched DataCite, Google Dataset Search and BASE in August 2021. The existing literature shows that many cytotoxic drugs, including methotrexate, a widely used drug for treatment of cancer and inflammatory diseases inhibit bacterial growth. Many of those drugs are also known to share molecular targets with commonly used antibiotics (e.g. methotrexate and trimethoprim). Further, it is proposed that cytotoxic drugs may drive antibiotic resistance evolution due to microbiome alterations, overlapping intrinsic resistome, and SOS induced mutagenesis.

**Added value of this study**

To our knowledge, we show for the first time that methotrexate directly selects for acquired trimethoprim resistance determinants on the chromosome as well as on a clinical multi-drug resistance plasmid. These selective and co-selective effects occur at methotrexate concentration ranges expected to represent intestinal concentrations during clinical use. Thus, we provide new evidence on how a cytotoxic drug can affect the evolution, selection, and spread of acquired antibiotic resistance determinants.

**Implications of all the available evidence**

The current antibiotic resistance crisis can have serious consequences for cancer treatment since these patients display higher risk of bacterial infections and consequently depend on antibiotic treatment. The indications that drugs used in cancer chemotherapy may drive resistance evolution through the same and/or similar resistance mechanisms as antibiotics is potentially of great concern for both cancer patients and the general society. This report represents a first step that will enable us to target drug combinations where resistance evolution is less likely to be an undesired side effect of cancer treatment.

Alt-text: Unlabelled box

## 1 Introduction

Global overuse and misuse of antimicrobial drugs in combination with dwindling discovery rates of new antimicrobials have led to the current antibiotic resistance crisis [1]. It is also increasingly clear that non-antibiotic natural and anthropogenic substances affect antibiotic resistance evolution in bacterial populations and exacerbates the problem. These include biocides, metals and non-antibiotic drugs that may either directly select for antibiotic resistance, play important roles as co-selective agents, influence horizontal gene transfer (HGT) or mutation rates, and potentiate the effect of low antibiotic concentrations [2], [3], [4], [5]. To effectively launch global initiatives to reduce antibiotic resistance there is an urgent need to identify novel drivers of resistance evolution. Antibiotic resistance is a major risk factor for patients with impaired immunity, such as cancer patients, and often a patient's survival depends on antibiotic treatment to reduce the risk for hospital-acquired infections during chemotherapy [6,7]. Several cytotoxic drugs used in cancer chemotherapy are known to both elevate bacterial mutation rates and have direct antimicrobial properties [8,9]. It has been proposed that cancer chemotherapy may drive *de novo* antibiotic resistance evolution through SOS induced mutagenesis [10], and some reports have provided support for this hypothesis [11,12]. Recently, the effects of non-antibacterial drugs on bacteria typically found in the human gut were thoroughly explored and cytotoxic drugs were reported to cause the most severe alterations of the microbiota [2]. Taken together, these studies suggest that cytotoxic drugs affect survival of human gut commensals, they may increase the evolvability of bacterial populations, and lead to reduced bacterial susceptibility towards drugs used to treat cancer. How bacterial populations respond to selective and co-selective pressures exerted by individual cytotoxic drugs and the implications for antibiotic resistance selection and spread is unknown. Thus, there is an urgent need to understand these potential collateral effects of cancer chemotherapy to ensure effective antibiotic treatment for a large group of immunocompromised patients. Moreover, cytotoxic drugs may constitute a previously unrecognized target for intervention to limit the selection and spread of antibiotic resistance.

Methotrexate (MTX) is widely used in treatments including but not limited to; cancer of the breast, skin, head, neck, and lung as well as many inflammatory diseases, such as rheumatoid arthritis [13]. We specifically targeted resistance towards trimethoprim (TMP), since both drugs are structurally similar (Figure S1) and act through inhibiting the dihydrofolate reductase enzyme in bacteria and eukaryotic cells, central in DNA synthesis [14]. TMP in combination with sulfamethoxazole is among the most frequently used antibiotics in the treatment of urinary tract infections and is recommended as first line treatment internationally [15]. Our main target organism in this study is *Escherichia coli*, the most common agent of nosocomial infections world-wide [16]. *E. coli* is known to display intrinsic resistance towards MTX through AcrAB-TolC mediated efflux [17], however TMP is not a substrate for this efflux system.

Previous studies have focused on the abilities of MTX and other non-antibiotics to inhibit bacterial growth [2]. These approaches have provided valuable insights on the effects of non-antibiotics as modulators of the intestinal flora, but lacked the necessary resolution to detect more subtle selective effects on acquired antibiotic resistance determinants.

Here, we hypothesize that despite the demonstrated *E. coli* intrinsic MTX resistance [17], MTX can affect antibiotic resistance evolution in *E. coli,* due to the shared molecular target with TMP. We show that MTX selects for acquired bacterial TMP resistance (TMP^R^) and co-selects for other antibiotic resistance determinants when co-residing on a mobile genetic element. Exposure to a wide concentration range of MTX selects for mutations identical to those emerging during TMP selection in clinical isolates of *E. coli*. Moreover, we show that the minimum selective concentrations (MSCs) of MTX and positive selection for chromosomal and plasmid-mediated TMP^R^ determinants occurs at concentrations 40- and >320-fold below the MTX minimum inhibitory concentrations (MICs), respectively.

## 2 Methods

### 2.1 Bacterial strains and growth conditions

Bacterial strains used in this study are listed in Table S1. All incubations of liquid cultures were performed with orbital shaking (225 rpm) at 37 °C, unless otherwise specified. Overnight cultures were grown in Miller Difco Luria-Bertani (LB) broth/agar (Becton, Dickinson and Co.). We used cation adjusted Mueller-Hinton II Broth (MHIIB, Becton, Dickinson and Co.) for assays with drugs supplemented to the media. When appropriate, media were supplemented with: 100 mg/L ampicillin (Sigma-Aldrich), 12.5 mg/L chloramphenicol (Sigma-Aldrich), 7.5 mg/L tetracycline (Sigma-Aldrich), 100 mg/mL Teva/Ebetrex (MTX) (Pharmachemie B.V./Ebewe Pharma Ges.m.b.H Nfg.KG). Methotrexate (MTX) was used in the form of a hydroxide solution ready for i.v. therapy. For strains harbouring the pBAD30 expression vector, cultures were supplemented with 0.2% (w/v) arabinose (Sigma-Aldrich) for induction. Generalized transduction using the P1*vir* [18] were used to move chromosomal markers between strains. For selection against cells expressing *sacB*, sucrose selection plates were used. For long-term storage, strains and populations were mixed with glycerol at a final concentration of 20% (v/v) and frozen at −80 °C.

### 2.2 Strain constructions

A promoter-levansucrase-chloramphenicol resistance-promoter cassette (P_CP25_-*sacB*-*cat*-P_J23101_) was first constructed by amplifying the *sacB*-*cat*-P_J23101_ cassette (GenBank: KM018298) by using primers with homologies to each end of the *insKJ* and partially *mokA* genes in the IS*150* region on the *E. coli* MG1655 chromosome (Table S2). The construct was introduced onto the chromosome by λ Red recombineering [19,20] in a strain carrying the pSIM5 plasmid [21] with tetracycline as the antibiotic selection marker (pSIM5-*tet*, DA45134). Chloramphenicol resistance was used to select for the inserted construct.

Fluorescent protein encoding: *bfp* (*cat*-PJ23101-*mtagBFP2*, blue; [4]; GenBank: KM018299), *yfp* (*cat*-PJ23101-*SYFP2*, yellow; [4]; GenBank: KM018300) were PCR amplified from previous strains [4]. PCR amplifications were carried out using Phusion High-Fidelity DNA Polymerase (Thermo Scientific). Reaction primers were designed with one of the 40 bp homology to the disrupted IS*150* locus whilst the other retained the P_CP25_ promoter (Table S2). Reaction products were purified using the GeneJet Purification Kit (Thermo Scientific) and introduced onto the chromosome by λ Red recombineering by counter-selection on sucrose agar medium. This resulted in [P_CP25_-*sYFP2*] and [P_CP25_-*mtagBFP2*] constructs.

Dup-In methodology of the IS*150* locus was carried out on all previous constructs [22] using the *sacB*-*cat*-P_J23101_ cassette (GenBank: KM018298) and chloramphenicol resistance as the antibiotic selection. P1*vir* lysates for both fluorescent markers were prepared and transduced into a common background (DA4201) by generalized transduction and segregation of Dup-Ins. Briefly, transduced colonies were picked from plates first with chloramphenicol resistance to transfer the Dup-In with the IS*150* locus, then single colonies were patched on sucrose plates for loss of the *sacB*-*cat*-P_J23101_ cassette but retaining of the IS*150* locus. For screening of the final strains constructed and generation of templates for Sanger sequencing, DreamTaq PCR Master Mix (Thermo Scientific) was used.

The fluorescently tagged strains were further engineered to obtain TMP^R^ derivatives. Two point mutations associated with *folA* (one in the *folA* gene, W30R, and the second 58 bp upstream of *folA* within the promotor region, C>T) were introduced onto the chromosome of the strains using a double MAGE cycle with the pORTMAGE-2 plasmid (RRID:Addgene_72,677) as described by previously [23]. The pG06-VIM-1 [24], was transformed into the fluorescently tagged strains as well as *Klebsiella pneumoniae* using room temperature electroporation [25].

To verify the role of *dfrA* genes in both TMP^R^ as well as methotrexate resistance (MTX^R^), both *dfrA1* and *dfrA12* were PCR amplified using Phusion High-Fidelity DNA Polymerase (New England BioLabs Inc.)(Table S2), purified using QIAquick PCR Purification Kit (QIAGEN), phosphorylated using T4 Polynucleotide Kinase (Thermo Scientific) and cloned using T4 DNA Ligase (Thermo Scientific) into the pBAD30 [26] vector at the *Sma*I site. Thus, gene expression was under a tightly inducible control by the P_BAD_ promotor when in the presence of arabinose [26]. The purified ligation reactions were transformed into electrocompetent DH5-α cells with electroporation and clones carrying the vector-born *dfrA* genes isolated.

### 2.3 Susceptibility testing

Due to the bacteriostatic activities of MTX and a lack of a gold standard for MTX microbiological assays we define the MIC of MTX in this study as the 90% inhibitory concentration (IC_90_). This allows for a high resolution and has previously been used as a proxy for the MIC [27,28]. The IC_90_ values for TMP and MTX were determined as described previously with minor changes [29]. Briefly, 96-well plates were incubated at 300 rpm when containing MTX and 700 rpm when containing TMP (3 mm stroke) for 18 h at 37 °C before the OD_600_ was measured using an Epoch 2 Microplate Spectrophotometer (BioTek Instruments, Inc.)/VersaMax™ ELISA Microplate (Molecular Devices®). Internal controls were included on all plates. Percent inhibition was calculated as previously described [30]. At least three biological replicates were used and the MIC was set as the most read (modal) value on a two-fold scale of replicates that met quality control standards. For characterization of TMP^R^ mutants isolated from the MTX sub-MIC evolution, TMP MIC was determined by gradient diffusion strips following the manufacturer's guidelines (Liofilchem). Measurements were done using two to four biological replicates, where the MIC was set as the most read (modal) value.

### 2.4 Growth rate measurements

Growth rates were determined using a Bioscreen C MBR reader (Oy Growth Curves Ab, Ltd). A minimum of five independent overnight cultures of each strain were diluted to ∼5 × 10^6^ CFU/mL in MHIIB containing MTX at concentrations ranging from 0 to 8 mg/mL. Two 300 μL aliquots of each dilution were transferred into sterile Honeycomb plates (Oy Growth Curves Ab, Ltd). The samples were grown at 37 °C with continuous shaking for 18 h and OD_600_ values were measured every 4 min. The growth curves from the Bioscreen C measurements were analysed and growth rate calculations done using the statistical software R [31]. In short, the R package Bioscreen Analysis Tool BAT 2.1 [32] was used to calculate the doubling time of each well by fitting a straight line to the logarithmic phase (OD_600_ values between 0.02 and 0.1). Relative growth rates were then calculated by dividing the mean doubling time of the reference strain grown without any drug present by the mean doubling time of the strain and condition being tested.

### 2.5 Competition experiments

Competition experiments were performed using the fluorescently tagged strain pairs, both for *folA* and pG06-VIM-1 mediated TMP^R^. A susceptible strain tagged with either *yfp* or *bfp* was mixed at 1:1 ratio with the constructed TMP^R^ strains harbouring the disparate fluorescence marker to initiate a head-to-head competition, at different MTX concentrations. Six independent cultures (∼5 × 10^9^ CFU/mL) of each strain were used to start 12 competitions, i.e. six biological replicates for each color arrangement in a dye-swap set-up. Every 24 h for three to four days the competing strains were passaged by a 1:1000 dilution into fresh medium and the mutant to wild type (wt) ratio measured by counting 10^5^ cells using a fluorescence-activated cell sorter (BD FACS Aria III). For safety reasons, all cultures were washed in fresh drug-free MHIIB in order to remove MTX from the cultures before FACS analysis. Cells were pelleted at 5000 rcf at 4 °C for 5 min, MTX containing supernatant removed and cells resuspended in fresh MHIIB.

Selection coefficients were calculated according to the regression model s=[*lnR(t)/R(0)*]/*t*, as previously described [33], where *R* is the mutant to wt ratio and t is the time measured in generations of growth. The minimum selective concentration (MSC) is defined as the concentration where the selection coefficient equals zero (where the regression line crosses the x-axis) [34].

In a similar way, six individual cultures of a susceptible *yfp* strain was competed against the *bfp* resistant strains in 1:1, 1:10, 1:10^2^, 1:10^3^ and 1:10^4^ starting ratios of TMP^R^:TMP^S^ strains at concentrations slightly above the estimated MSCs (400 μg/mL for *folA* mutant, 75 μg/mL for p06-VIM-1).

To assess the stability of the pG06-VIM-1 in the presence of MTX, three independent lineages of K56–75 harbouring the plasmid (MP05–31) were serially passaged for 50 generations (1:100-dilution) in 1 mL MHII batch cultures with 400 μg/mL MTX. The lineages were then plated on non-selective agar. One hundred colonies from each lineage was replica plated and reduced susceptibilities towards ampicillin, TMP, streptomycin and spectinomycin determined by patching on MHII agar supplemented with 100 μg/mL ampicillin, 25 μg/mL TMP, 40 μg/mL streptomycin or 40 μg/mL spectinomycin as well as MHII agar.

### 2.6 Selective plating on high concentrations of MTX

Single MTX resistant mutants of K56–2 (MP06–01) were selected at lethal MTX concentrations. Dense overnight cultures grown in drug-free LB was concentrated 10 ×, and 100 μL spread on LB agar plates supplemented with 4, 8 and 16 mg/mL MTX. Mutants were picked after 48 to 96 h and purified on non-selective plates. Additionally, an overnight culture in LB containing MTX at the estimated MIC concentration was concentrated 10 × and 100 μL spread on LB agar plates with and without MTX 32 mg/mL. After 48 h incubation, mutants were purified on non-selective plates. The MTX and TMP MICs for all mutants isolated were determined as previously described by IC_90_ testing [29] and the *folA* gene, its promotor area and the *marR* gene sequenced with Sanger sequencing and analyzed using the CLC Main Workbench (QIAGEN).

### 2.7 Laboratory evolution at sub-MICs of MTX

To examine the effect of MTX on TMP^R^ evolution, strain K56–2 (MP06–01) was serially passaged in liquid cultures with MTX supplemented at concentration slightly above the estimated MSC. Initially, 10 independent overnight cultures were started from independent colonies on separate agar plates from which ∼10^3^ cells were used to start ten independent lineages in 1 mL MHIIB containing 400 μg/mL MTX (lineages 1–10). Every 12 h for 25 days, the lineages were serially passaged by 1000-fold dilution in 1 mL batch cultures, allowing for ∼500 generations of growth. Every ∼50 generations the populations were frozen down at –80 °C. In parallel, three independent control lineages were simultaneously sampled for TMP resistance under the same experimental conditions except for MTX exposure (lineages 11–13). After ∼500 generations of growth end-point populations were plated on MHII agar plates containing 32 mg/mL MTX. From lineages 1–10, 20 colonies were isolated from each and tested for TMP^R^ with no increase in TMP resistance detected compared to the parental strain. The frozen populations were gently thawed on ice and dilution series plated on both MHII agar with TMP 4 μg/mL and without drug, and frequencies of TMP resistant mutants calculated. From each plate where mutants grew, up to five colonies were randomly isolated, their susceptibility towards TMP measured, and the *folA* gene and its promotor area sequenced with Sanger sequencing and analysed using the CLC Main Workbench (QIAGEN). No MTX^R^ or TMP^R^ colonies were isolated from the lineages grown without drug (lineages 11–13).

### 2.8 Whole genome sequencing

To investigate the possibility of additional genetic changes during MTX selection, other than the TMP^R^ determinants shown to be associated with reduced susceptibility towards MTX, five isolates from the lethal selection were chosen (MP18–13, MP18–17, MP18–20, MP18–26 and MP18–28) based on their different susceptibility profiles and subjected to whole genome sequencing (WGS). Bacteria were grown overnight and genomic DNA prepared using GenElute™ Bacterial Genomic DNA Kit (Sigma-Aldrich) following the manufacturer's instructions with slight adaptions. In brief, 1.5 mL of dense culture (OD_600_: 0.8–1.0) was pelleted by centrifugation at 13000 rpm and supernatant removed. The pellet was resuspended in 200 μL lysozyme solution (100 mg/mL) and incubated for 30 min at 37 °C, before 20 μL of RNase A solution was added and incubated for 2 min at room temperature. Following, 20 μL of Proteinase K (20 mg/mL) and 200 μL of Lysis solution C were added to the mixture and incubated at 55 °C for 10 min after being thoroughly vortexed. To each pre-assembled GenElute Miniprep Binding Column, 500 μL of the Column Preparation Solution were added, 200 μL of ethanol (95–100%) was then added to the lysate and thoroughly mixed before the lysate was carefully loaded onto the binding column, centrifuged at 13000 rpm for 1 min and then washed 2 × with 500 μL of Wash Solution. Genomic DNA was eluted in 100 μL of 10 mM Tris-base and purity and concentration determined using NanoDrop One (Thermo Scientific) and Qubit (Thermo Scientific) respectively. Next-generation sequencing libraries were prepared from the bacterial genomic DNA samples and sequenced on an Illumina NovaSeq with a 2 × 150 bp configuration (GENEWIZ). Average whole genome coverage per sample was approximately 700. Analysis of the fastq files obtained from Illumnia sequencing was performed using an in-house bioinformatic pipeline (Table S3) to compare the mutant sequences to the previously published wt strain (available at NCBI, BioSample SAMN08095529). Where single-nucleotide polymorphisms (SNPs) were identified with a coverage below 100, the evidence was considered insufficient and the SNPs were removed from the analysis. Raw sequence reads were deposited under BioProject PRJNA677979.

### 2.9 Statistics

Means and standard deviations were estimated using the software R (version 4.1.0) and RStudio (version 1.4.1717).

### 2.10 Role of the funders

The funders had no role in study design, data collection, data analysis, interpretation, or writing of the report.

## 3 Results

### 3.1 Methotrexate selects for pre-existing TMP^R^ determinants

We initially determined the MICs of MTX in clinical [35] and laboratory strains of *E. coli* (Table S1). Initial experiments revealed variable, but high MTX MICs, ranging from 4 to 32 mg/mL in the different genetic backgrounds, with the exception of <0.25 mg/mL for *E. coli* W3110 Δ7NR*tolC* (Tables S4-S5) [36]*.* This being consistent with previous reports demonstrating that *E. coli* displays intrinsic resistance towards MTX due to AcrAB-TolC mediated efflux [2,17]. We also observed that the MTX MIC was dependent on the presence of TMP^R^ determinants. All isolates with a functional TMP^R^ determinant and increased TMP MIC showed consistently higher MTX MICs (>32 mg/mL) than TMP susceptible (TMP^S^) isolates (4–32 mg/mL), indicating possible co-selective abilities of the two drugs. This included strains of both *E. coli* as well as *K. pneumoniae* ATCC13883 harbouring the clinical multi-drug resistance (MDR) plasmid pG06-VIM-1. Strains of both species harbouring the plasmid displayed reduced susceptibility towards MTX as well as TMP (Table S4-S5).

Antibiotic resistance selection and co-selection have traditionally been assumed to occur between the MICs of susceptible and resistant isolates within a bacterial population (known as the selective window) [34]. However, several reports unequivocally show that antibiotic resistance selection and co-selection can occur at concentrations several hundredfold below the MIC of a susceptible isolate (known as sub-MIC) [4,34,37]. To test how sub-MICs of MTX affect bacterial fitness, we measured exponential growth rates for two pairs of clinical isogenic TMP^R^ and TMP^S^ *E. coli* across a wide MTX concentration span. One pair with TMP^R^ located on the chromosome (one intragenic point mutation *T*>*A* (W30R) in the *folA* gene, and one in its promotor region (P*_folA_*, C>T 58 base pairs (bp) upstream of the gene)(MP06–01) [29] and one pair with TMP^R^ (*dfrA*) located on the MDR plasmid pG06-VIM-1 (MP05–31)(24). TMP^S^ strains displayed sharply declining growth rates between 1 and 2 mg/mL of MTX, whereas the TMP^R^ strains remained unaffected (Figure S2, Table S6). These results suggest a selective benefit during MTX exposure for TMP^R^ strains at concentrations below the observed MTX MIC of the TMP^S^ clinical isolates. The same effect was observed in the nosocomial pathogen *K. pneumoniae* ATCC13883 where a dose response curve comparing the strain with and without pG06-VIM-1 shows a clear difference in susceptibility already at concentrations below 2 mg/mL (Figure S3, Table S7-S8).

**Fig. 1:**
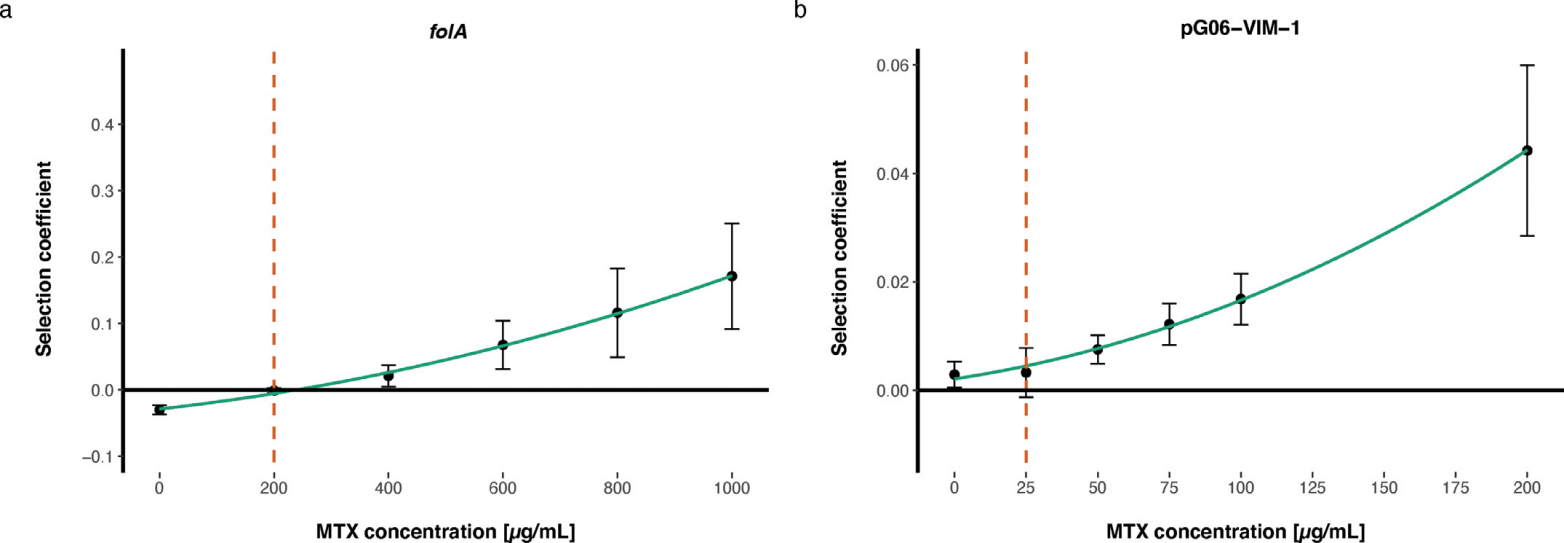
**Selection coefficients as functions of MTX concentrations from competition experiments between TMP**^**R**^**and TMP**^**S**^**isogenic strains.** The MSC is defined as the concentration where the selection coefficient equals zero. The MSC of *E. coli* MG1655 harboring (a) two chromosomal *folA* mutations (MP18–04 and MP18–07) is set to 200 μg/mL, and (b) the MDR pG06-VIM-1 plasmid encoding *dfrA12* (MP18–05 and MP18–08) is conservatively set at 25 μg/mL. Dashed lines represent the set MSC, bullets the average selection coefficients based on 12 individual replicates and error bars the standard deviations.

The MSC describes pharmacodynamically the lowest concentration where selection for resistance occurs [34]. To determine the MSC for MTX, we constructed a fluorescently tagged pair of *E. coli* MG1655 strains to enable accurate separation between the two in mixed populations. In these backgrounds, we introduced TMP^R^, either through mutations (*folA*) using genome engineering or the pG06-VIM-1 plasmid. The isogenic TMP^R^ and TMP^S^ strain pairs were competed head-to-head by serial passage for 30 generations and the ratio of TMP^R^:TMP^S^ was determined over time using flow cytometry. From this data the MSC was estimated (Tables S9-S10 [34]. Chromosomal *folA* mutations reduced fitness in *E. coli* MG1655 with 3.01% (+/- 0.71, SD) (Table S9) and displayed an MSC of 200 μg/mL (1/40 of the MIC of MTX) (Fig. 1). The MDR plasmid pG06-VIM-1 was selectively neutral (potentially slightly beneficial) displaying a 0.29% (+/- 0.24, SD) increase in fitness (Table S10). The latter estimates of relative fitness were close to the detection limit of the assay [34], and we conservatively estimated the MSC to be <25 μg/mL (less than 1/320 of the MIC of MTX) (Fig. 1). Taken together, our data strongly suggest that selection for TMP^R^ occurs at MTX concentrations far below the estimated MTX MIC.

### 3.2 Sub-MICs of methotrexate promotes invasion of TMP^R^ determinants even when rare in *E. coli* populations

**Fig. 2:**
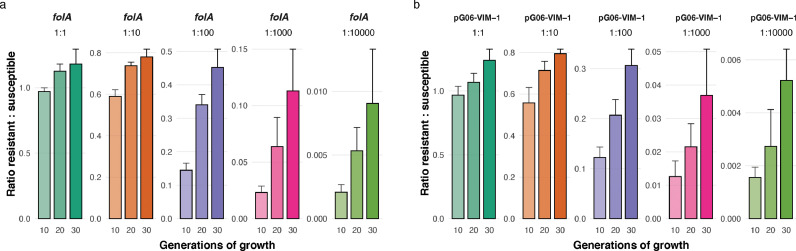
**Competition experiments during sub-MIC MTX exposure at different initial frequencies of TMP**^**R**^**strains.** The change in TMP^R^:TMP^S^ ratios over 30 generations of growth at MTX concentrations slightly above the MSCs where *E. coli* MG1655 harbors (a) two *folA* mutations (MP18–07), and (b) the MDR pG06-VIM-1 plasmid (MP18–08) were competed against a differently tagged, isogenic susceptible strain (DA56507) at 1:1, 1:10^−2^, 1:10^−3^ and 1:10^−4^ starting ratios. Error bars represent the standard deviation of the average ratio, based on 6 individual replicates.

Exploring MTX-selective dynamics further, we asked if TMP^R^ determinants could invade the population at lower initial densities to exclude potential bias from the 1:1 ratio in the competition experiments. We started competition experiments from frequencies as low as 10^−4^ of the TMP^R^ strains, at concentrations slightly above the estimated MSC of MTX (400 μg/mL for *folA* mediated resistance and 75 μg/mL for pG06-VIM-1 mediated resistance) and followed the change in ratios over 30 generations of growth (Fig. 2, Table S11). Both chromosomal and plasmid mediated TMP^R^ determinants were able to invade, even when initially rare in their respective populations, strongly suggesting that the MTX selective effects are independent on initial frequencies of resistant and susceptible strains during competition experiments.

### 3.3 Methotrexate co-selects for resistance determinants on a multi-drug resistance plasmid

The *dfr*-genes represent a common TMP^R^ mechanism in *E. coli* and these genes are frequently located on mobile genetic elements such as integrons and plasmids. Given that MTX selects for *dfr*-mediated TMP^R^, co-selection of other genetically linked resistance genes is likely. To show this, we used the MDR pG06-VIM-1 plasmid harboring *dfrA1* and *dfrA12* along with multiple resistance determinants including four aminoglycoside resistance genes and the *bla*_VIM-1_ carbapenemase gene conferring resistance to broad-spectrum β-lactams including carbapenems [24]. To assess the stability of pG06-VIM-1 in our strains competing in the presence of MTX, *E. coli* K56–75 harboring the plasmid (MP05–31) was serially passaged in batch cultures with 400 μg/mL MTX supplemented for 50 generations. The lineages were then plated on non-selective agar and 100 colonies from each lineage tested for reduced susceptibility towards ampicillin, TMP, streptomycin and spectinomycin. The results revealed complete phenotypic stability across all three lineages, confirming MTX mediated co-selection of plasmid-mediated MDR.

**Fig. 3:**
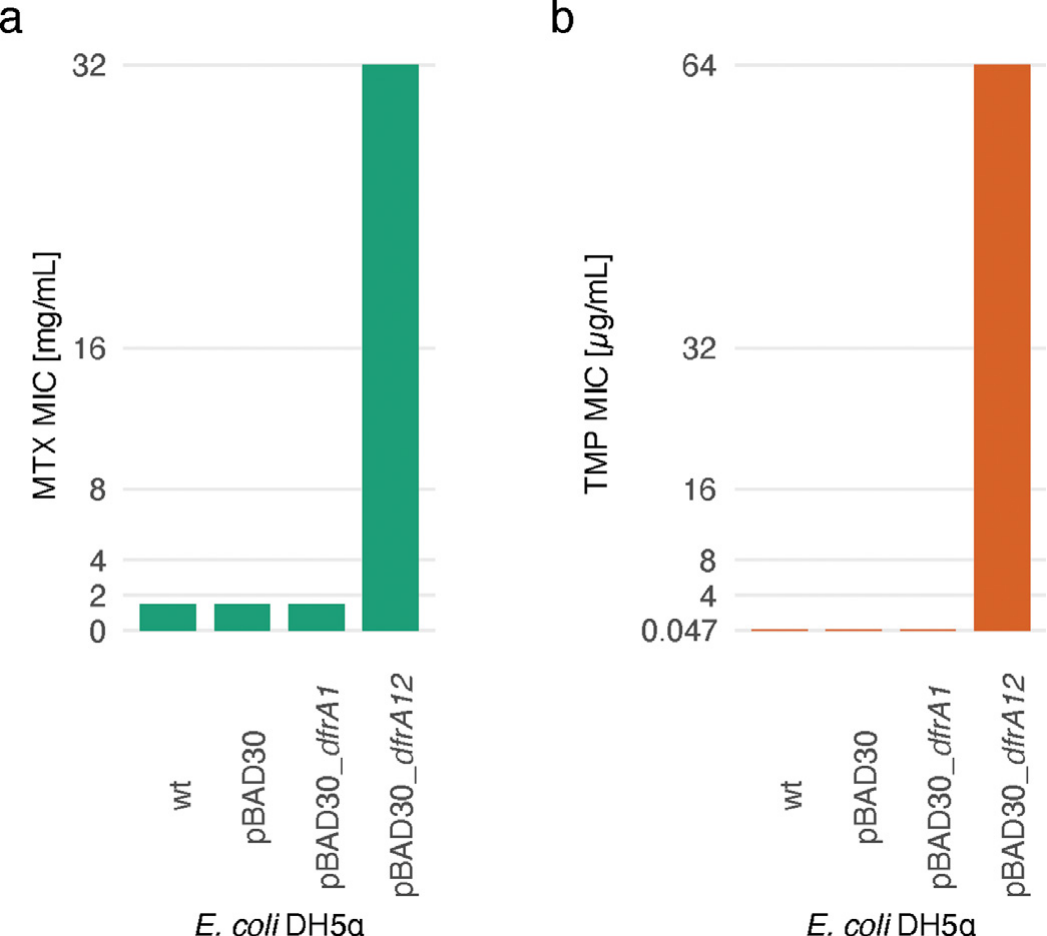
**MTX and TMP MIC of*****E. coli*****DH5α expressing*****dfrA1*****or*****dfrA12.*** The MTX (a) and TMP (b) MIC for the wild type (wt) *E. coli* DH5α (MP18–09) compared to the strain harboring the empty pBAD30 (MP18–10) expression vector as well as strains with pBAD30 with different *dfrA* genes expressed under the inducible expression control by the pBAD promotor (MP18–11 and MP18–12). The detection limit of the assay is 64 μg/mL for TMP and 32 mg/mL for MTX. For both drugs, the MIC of *E. coli* DH5α expressing *dfrA*12 (MP18–12) exceeded the detection limit whereas the strain expressing *dfrA1* (MP18–11) has the same MICs as the wt strain. Showing that MTX and TMP resistance conferred by the pG06-VIM-1 plasmid is caused by the *dfrA12* gene.

To verify that the TMP^R^ determinants on the MDR pG06-VIM-1 plasmid is the primary mediators of MTX resistance and selection, both *dfrA1* and *dfrA12* were isolated from the plasmid (Table S2) and cloned onto an expression vector and the effects of the individual genes measured. Of the two genes, only *dfrA12* was shown to give the same resistance pattern for TMP as well as MTX as the pG06-VIM-1 plasmid (Fig. 3), and the lack of detectable phenotype for *dfrA1* (MP18–11) is likely due to a start codon frameshift mutation [24].

### 3.4 Methotrexate selects for *de novo* TMP^R^

We further examined whether exposure to MTX could lead to *de novo* TMP^R^ evolution. We selected spontaneous mutants from overnight cultures with and without exposure to MTX, plated on selective agar at high MTX concentrations and tested for TMP cross-resistance (Figure S4, Table S12). *E. coli* K56–2 isolated at 16 and 32 mg/mL MTX (MP18–17 to MP18–28) displayed increased MICs of TMP close to or above the clinical breakpoint [38], clearly demonstrating selection for TMP^R^ by MTX. DNA sequencing of the resistant isolates revealed two different mutations in the *folA* promoter, previously reported to result in TMP^R^ [39], as well as a single mutation in the *marR* gene (Tables S12-S17).

**Fig. 4:**
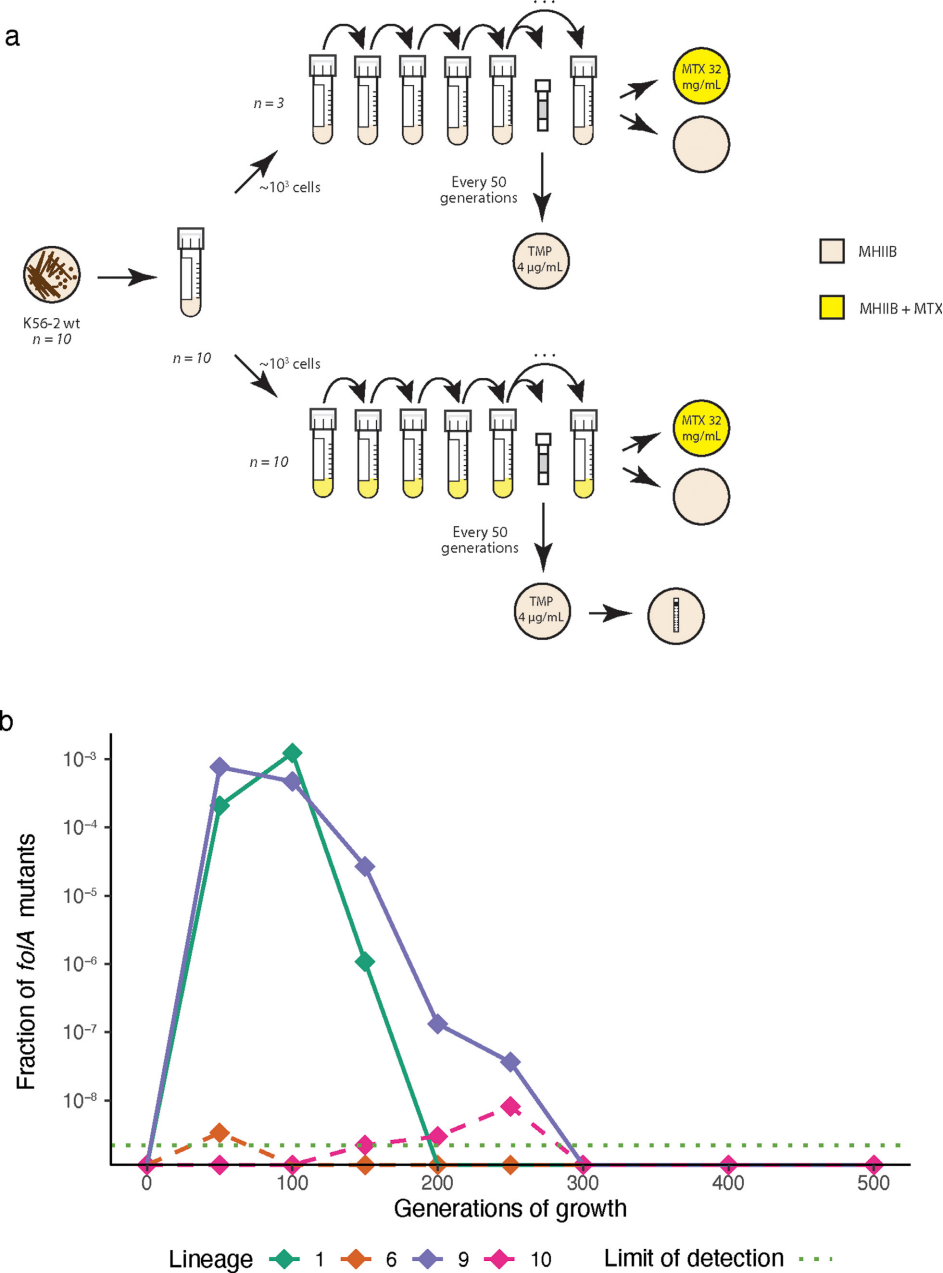
**Evolution of TMP**^**R**^**during MTX exposure for 500 generations.** (a) Sub-MIC evolution experimental set-up. Ten biological replicates of K56–2 (MP06–01) were evolved for ∼500 generations with 400 μg/mL MTX and three biological replicates without drug. All lineages were screened for TMP^R^ every 50 generations. After 500 generations all end-point populations were plated on 32 mg/mL MTX. All populations were able to grow at 32 mg/mL MTX, but not a single clone isolated conferred TMP^R^, strongly suggesting that reduced susceptibility to MTX with no cross-resistance to TMP evolved in the endpoint populations. (b) Fractions of TMP^R^*folA* mutants isolated every 50 generations from the lineages where these were detected. The detection limit of the assay was ∼2 × 10^−9^. Solid lines represent the two lineages where TMP^R^ emerged and ascended in frequency whereas dotted lines indicate spontaneous mutants.

Finally, we asked if exposure to sub-MICs of MTX close to the estimated MSCs would select for *de novo* TMP^R^ mutations in a susceptible *E. coli* population. Starting from 1000 cells to minimize the probability of pre-existing mutants, we grew ten independent lineages of the *E. coli* K56–2 strain at 400 μg/mL MTX for 500 generations. The frequency of TMP^R^ was determined every 50 generations. TMP^R^ ascended in frequency in 2/10 lineages at different rates and time-points during the first 250 generations before they were outcompeted by a different set of mutants with reduced susceptibility to MTX and no cross-resistance to TMP (Fig. 4, Table S18). These experiments show that MTX exposure can select for *de novo* TMP^R^, both at high and sub-MIC concentrations. Arguably, the emergence of *folA* mutations in only 2 lineages is likely due to a larger mutational target within AcrAB-TolC, resulting in reduced susceptibility towards MTX (and not TMP).

### 3.5 Pharmacokinetic approximations

To assess pharmacokinetic relevance, we attempted to estimate the MTX concentration range likely to be found in the intestine of patients undergoing MTX treatment. Limited information is available on gut MTX concentrations following intravenous administration during cancer treatment, as pointed out by others [2]. Pharmacokinetic data reveal that up to 90% of administered MTX is renally excreted [40] and we assume that the remaining ∼10% of the dose constitutes the upper limit of the concentration range present in the human intestine. The lower limit is set to 2% of the dose based on the mean ^3^H labelled MTX concentrations measured in stool samples from nine patients receiving MTX intravenously [41]. From this, we set a 24 hour transition time in a total volume of 0.6 L [2] and calculated the dose (*d*) required to achieve MSC in the human intestine from:

$$\frac{d}{0.6L}\;x\;\left(0.1\;or\;0,02\right)=MSC$$

Estimated doses needed to reach intestinal MSCs assuming 2% and 10% fecal MTX concentrations were from 0.15 g to 0.75 g for plasmid-mediated TMP^R^ and from 1.2 g to 6 g for chromosomal *folA* mutations. Thus, assuming close to 2m^2^ body surface in grown-up patients [42] estimates of MSC for plasmid-mediated TMP^R^ translates to dosing regimens from 75 to 375 mg/m^2^ and from 0.6 to 3 g/m^2^ for the chromosomal *folA* mutations. These approximations indicate that our MSC estimates are relevant for patients receiving high dose MTX treatment (1–12 g/m^2^) [43]. A recent study, also using a literature-based approach but with slight differences, estimates gut MTX concentrations following oral administration during treatment of rheumatoid arthritis [44]. Their data suggested MTX concentrations as high as 100 μg/mL are found in the lower intestine, suggesting that our estimated MSC for plasmid-mediated antibiotic resistance determinants (25 μg/mL) is well within this concentration range.

## 4 Discussion

Here we show that exposure to the cytotoxic drug MTX affects selection and evolution of TMP^R^ determinants at clinically relevant concentrations. Notably, MTX can mediate selection of any antibiotic resistance determinant in *E. coli* when TMP^R^ is co-localized on a mobile genetic element across a wide concentration gradient. Transferring the MDR plasmid pG06-VIM-1 into a *K. pneumoniae* strain resulted in reduced susceptibility towards MTX, suggesting that our findings are relevant beyond *E. coli*. Arguably, this potentially important side-effect of MTX treatment has been previously unrecognized, as studies on the effects of non-antibiotic drugs, including MTX, have either focused on bacterial growth inhibition or used drug concentrations around the MIC [2,[45], [46], [47]], with a few exceptions [4,48].

Using the approaches outlined here, including high resolution mixed culture competition experiments, allow determination of the true MTX selective window ranging from the MSC to the MIC [34]. This is particularly relevant for non-antibiotics for which bacteria display reduced susceptibility. In *E. coli,* MTX is a substrate for the AcrAB-TolC efflux pump [17] and selective effects as those demonstrated here would not have been detected in classical susceptibility and/or growth assays in bacterial monocultures. This was recently supported in an *E. coli* chemical genetic screen where clear growth inhibitory effects of MTX, as well as for a range of other non-antibiotics, were only demonstrated in a *tolC* knock-out mutant (i.e. in a mutant lacking the intrinsic mechanism of resistance) [2].

Given that many cytotoxic drugs are structurally similar to antibiotics (e.g. doxorubicin/tetracyclines), or target similar key processes as the major antibiotic groups (e.g. DNA/protein synthesis) it is possible that cancer chemotherapy may lead to increased levels of antibiotic resistance in a vulnerable patient group that very often rely on efficient antibiotic treatment for survival. To acquire a deeper understanding of the evolutionary potential of novel, non-antibiotic drivers of antibiotic resistance the approaches presented here are essential. These approaches need to be combined with an improved understanding of the intestinal pharmacokinetics of MTX and other cytotoxic drugs, possible effects of co-administered drugs such as leucovorin mediated MTX rescue [49], and their interactions with the human microbiome. Such knowledge could allow identification of antibiotic + non-antibiotic drug combinations that should be avoided to preempt resistance evolution. This would be particularly relevant when considering repurposing cytotoxic drugs as antibiotics [50].

Taken together with recent studies showing that non-antibiotics can increase mutation rates [11,12] and promote horizontal gene transfer [48,51], the data presented here strengthens the evidence that non-antibiotic drugs can affect the evolution, selection, and spread of antibiotic resistance determinants. Our study is however not without limitations. Despite our pharmacokinetic considerations, which suggest that MTX selects and co-selects for antibiotic resistance determinants at clinically relevant concentrations, the lack of clinical data does limit our ability to conclude on the clinical and physiological significance of the results. Carefully designed *in vivo* experiments and/or clinical patient studies are important next steps to verify how MTX affect evolution, selection and spread of TMP resistance. One such approach could be a case control study comparing antibiotic resistance levels in patients that receive MTX compared to a group that does not, followed by microbiological and molecular analyses of bacteria and resistance determinants.

In this study we present data suggesting that MTX, a widely used drug in the treatment of several cancers as well as inflammatory diseases, may affect the evolution, selection and spread of antibiotic resistance. Moreover, we present an experimental frame-work where the true selective windows of non-antibacterial drugs can be determined. We argue that these approaches are critical to improve our understanding of non-antibacterial drugs as potential drivers of antibiotic resistance.

## 5 Contributors

The study was designed by JSG, EGAF, ØS and PJJ. Strain constructions were done by PT and JSG. Experiments were conducted by JSG, EGAF and CIMK. WGS analysis was done using a bioinformatic pipeline designed by JH. WGS data was verified by JH and JSG. Underlying data was verified by JSG and PJJ. Formal data analysis and data visualization was done by JSG. The first draft of the manuscript was written by JSG and revised by PJJ. All authors revised subsequent version and provided key edits to the manuscript. All authors read and approved the final version of the manuscript. Funding acquisition and resources were provided by PJJ and DIA.

## Declaration of Competing Interest

The authors declare no competing interests.
