# Supplementary material for: The chemotherapeutic drug methotrexate selects for antibiotic resistance
Source: eBioMedicine. 2021 Dec 11;74:103742. doi: 10.1016/j.ebiom.2021.103742 (PMC8671861; doi:10.1016/j.ebiom.2021.103742)
Supplement: Supplementary file 1 [file mmc1.docx]

**Supplementary Figures**

**
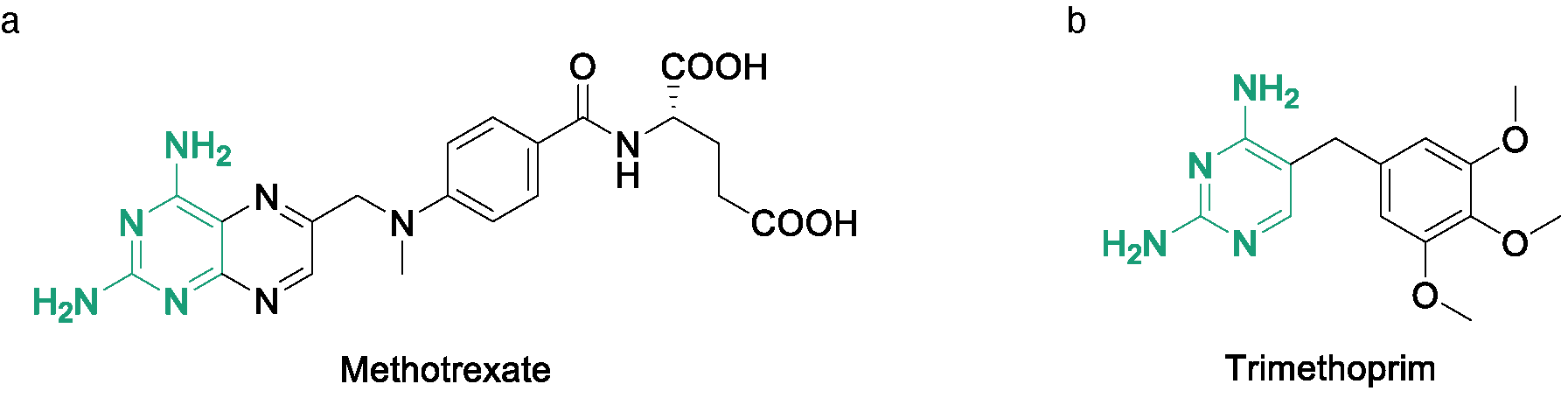
**

**Figure S1: Comparison of the chemical structures of (a) MTX and (b) TMP.** Both drugs are antifolates, that target and bind to the dihydrofolate reductase, and share a common 2,4-diaminopyrimidine moiety (highlighted in green).

**
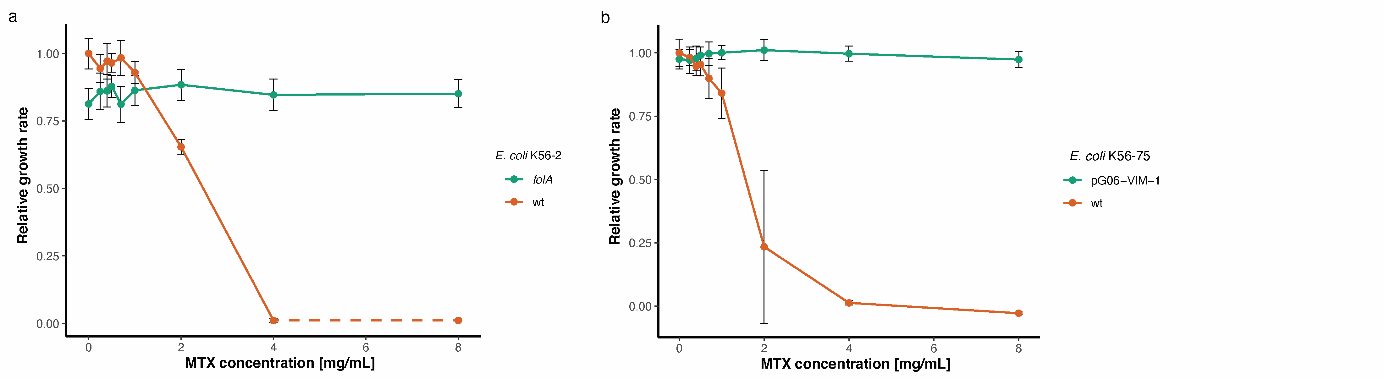
**

**Figure S2:** **Relative growth rates of TMP^R^ strains compared to their susceptible WTs as functions of MTX concentration. (a)** Chromosomally mediated TMP^R^ through *folA* mutations allows the K56-2 (MP06-05) strain to remain unaffected at MTX concentrations as high as 8 mg/mL whereas the TMP susceptible WT strain (MP06-01) shows a steep decrease in relative growth rates already between 1 and 2 mg/mL MTX. As we are close to the MIC of MTX for the TMP^S^ strain at 8 mg/mL MTX, growth rate measurements were difficult to interpret due to noise in the measurements. Dotted line indicate that the value is set close to zero. **(b)** Plasmid-mediated TMP^R^ through *dfrA* located on pG06-VIM-1 (MP05-31) allows the K56-75 strain to remain unaffected at MTX concentrations as high as 8 mg/mL whereas the TMP susceptible WT strain (MP06-41) shows a steep decrease in relative growth rates already at 0·7 mg/mL MTX. The error bars represent the standard deviation of the mean growth rate of 10 replicates (5 biological replicates with 2 technical replicates each).

**
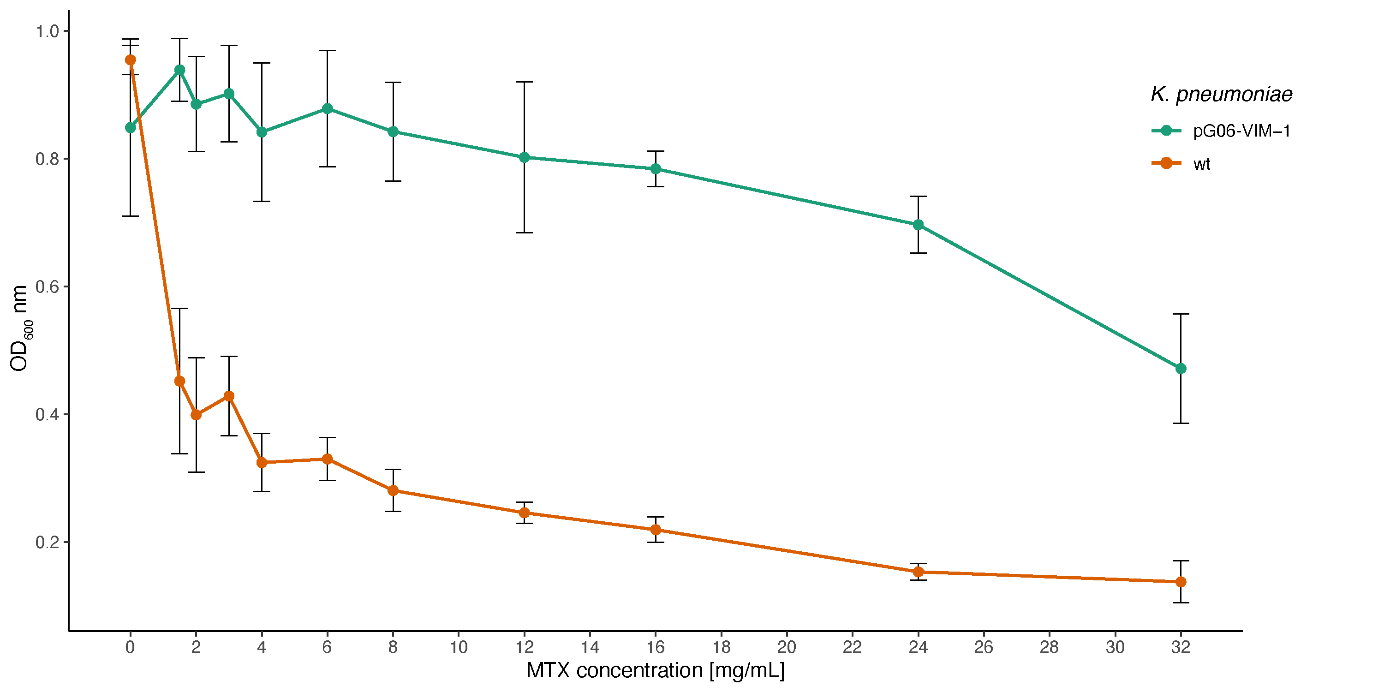
**

**Figure S3:** **Dose response curves of *Klebsiella* *pneumoniae* ATCC13883 with and without the pG06-VIM-1 plasmid.** The optical desnity at 600 nm (OD_600_) was measured following 18 hour shaking incuation of *K. pneumoniae* harbouring the pG06-VIM-1 plasmid (orange line) (MP26-52) and respective parent strain without the plasmid (blue line) (MP08-03) at varying concentrations of MTX. Points represent the average of three biological replicates and error bars indicate standard deviation. At the upper limit of detection, 32 mg/mL, there is a clear difference in the susceptibility of the isolate with and without the pG06-VIM-1 plasmid.


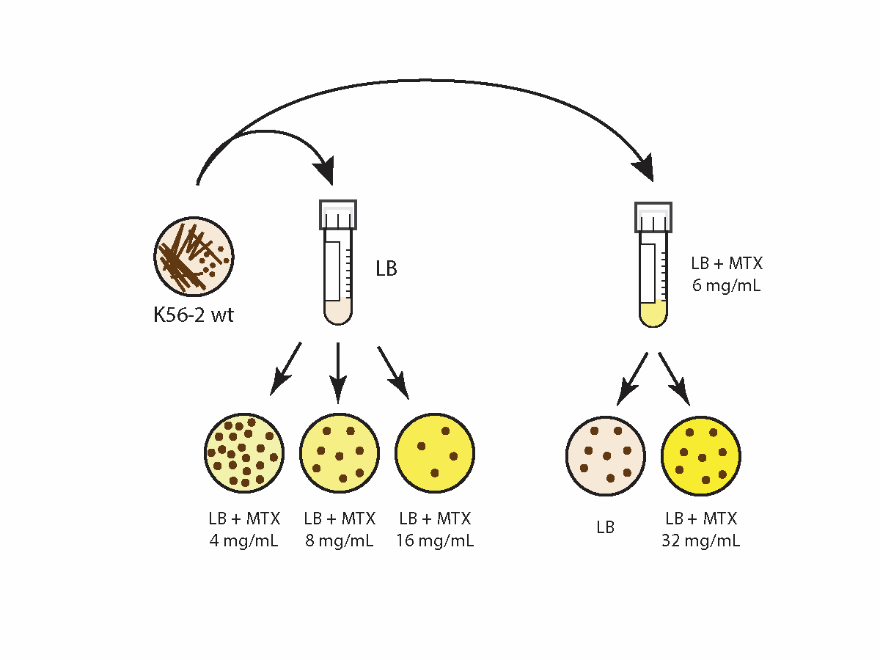


**Figure S4:** **Schematic of the experimental set-up used during selective plating at high MTX concentrations.** The MTX susceptible K56-2 strain (MP06-01) was grown overnight in LB without any drug present before plating on increasing concentrations of MTX, 4, 8 and 16 mg/mL. In a similar manner the K56-2 strain (MP06-01) was grown in LB + 6 mg/mL MTX before being plated on both selective (LB + MTX 32 mg/mL) and non-selective media. Clones isolated from all plates were re-streaked on non-selective media before being frozen down for characterization.

**Supplementary Tables**

**Table S1:** List of bacterial strains used and constructed for this study.

| **Strain** | **Genotype** | **Reference** |
| --- | --- | --- |
| DA4201 (MP13-01)/DA5438 | Wild type *E. coli* MG1655 (parent) | DA Strain Collection^1^ |
| DA45134 | DA5438, ∆IS*150*::*yetiYFP* pSIM5-*tet* | DA Strain Collection^1^ |
| DA52562 | DA45134, ∆IS*150*::*sacB*-*cat* | This study |
| DA52626 | DA52562, ∆IS*150*::*mTagBFP2* pSIM5-*tet* | This study |
| DA55343 | DA52562, ∆IS*150*::*sYFP2* pSIM5-*tet* | This study |
| DA55288 | DA52626, dupl[∆IS*150*::*mTagBFP2*]**cat*-*sacB* pSIM5-*tet* | This study |
| DA55290 | DA55343, dupl[∆IS*150*::*sYFP2*]**cat*-*sacB* pSIM5-*tet* | This study |
| DA56507 (MP18-01) | DA4201, ∆IS*150*::*sYFP2* | This study |
| DA52848 (MP18-02) | DA4201, ∆IS*150*::*mTagBFP2* | This study |
| DA12755 (MP08-03) | *K. pneumoniae* ATCC13883 | DA Strain Collection^1^ |
| MP05-31 | MP06-41 p06-VIM-1 | (24) |
| MP06-11 | *E. coli* K56-16 ST127 | (35) |
| MP06-16 | *E. coli* K56-41 ST73 | (35) |
| MP06-21 | *E. coli* K56-44 ST12 | (35) |
| MP06-26 | *E. coli* K56-50 ST100 | (35) |
| MP06-31 | *E. coli* K56-68 ST95 | (35) |
| MP06-36 | *E. coli* K56-70 ST537 | (35) |
| MP06-41 | *E. coli* K56-75 ST69 | (35) |
| MP06-46 | *E. coli* K56-78 ST1235 | (35) |
| MP18-03 | DA56507, C>T 58 bp upstream of *folA* | This study |
| MP18-04 | DA56507*,* W30R, C>T 58 bp upstream of *folA* | This study |
| MP18-05 | DA56507 pG06-VIM-1 | This study |
| MP18-06 | DA52848, C>T 58 bp upstream of *folA* | This study |
| MP18-07 | DA52848, W30R, C>T 58 bp upstream of *folA* | This study |
| MP18-08 | DA52848 pG06-VIM-1 | This study |
| MP18-09 | *E. coli* DH5α | Thermo Scientific |
| MP18-10 | MP18-09 pBAD30 | This study |
| MP18-11 | MP18-09 pBAD30_*dfrA1* | This study |
| MP18-12 | MP18-09 pBAD30_*dfrA12* | This study |
| MP18-13 | MP06-01, mutant isolated during MTX selective plating | This study |
| MP18-14 | MP06-01, mutant isolated during MTX selective plating | This study |
| MP18-15 | MP06-01, mutant isolated during MTX selective plating | This study |
| MP18-16 | MP06-01, mutant isolated during MTX selective plating | This study |
| MP18-17 | MP06-01, mutant isolated during MTX selective plating | This study |
| MP18-18 | MP06-01, mutant isolated during MTX selective plating | This study |
| MP18-19 | MP06-01, mutant isolated during MTX selective plating | This study |
| MP18-20 | MP06-01, mutant isolated during MTX selective plating | This study |
| MP18-21 | MP06-01, mutant isolated during MTX selective plating | This study |
| MP18-22 | MP06-01, mutant isolated during MTX selective plating | This study |
| MP18-23 | MP06-01, mutant isolated during MTX selective plating | This study |
| MP18-24 | MP06-01, mutant isolated during MTX selective plating | This study |
| MP18-25 | MP06-01, mutant isolated during MTX selective plating | This study |
| MP18-26 | MP06-01, mutant isolated during MTX selective plating | This study |
| MP18-27 | MP06-01, mutant isolated during MTX selective plating | This study |
| MP18-28 | MP06-01, mutant isolated during MTX selective plating | This study |
| MP19-78 | MP06-01, mutant isolated during MTX sub-MIC evolution | This study |
| MP19-79 | MP06-01, mutant isolated during MTX sub-MIC evolution | This study |
| MP19-80 | MP06-01, mutant isolated during MTX sub-MIC evolution | This study |
| MP19-81 | MP06-01, mutant isolated during MTX sub-MIC evolution | This study |
| MP20-01 | MP06-01, mutant isolated during MTX sub-MIC evolution | This study |
| MP20-02 | MP06-01, mutant isolated during MTX sub-MIC evolution | This study |
| MP20-03 | MP06-01, mutant isolated during MTX sub-MIC evolution | This study |
| MP20-04 | MP06-01, mutant isolated during MTX sub-MIC evolution | This study |
| MP20-05 | MP06-01, mutant isolated during MTX sub-MIC evolution | This study |
| MP20-06 | MP06-01, mutant isolated during MTX sub-MIC evolution | This study |
| MP20-07 | MP06-01, mutant isolated during MTX sub-MIC evolution | This study |
| MP20-08 | MP06-01, mutant isolated during MTX sub-MIC evolution | This study |
| MP20-09 | MP06-01, mutant isolated during MTX sub-MIC evolution | This study |
| MP20-10 | MP06-01, mutant isolated during MTX sub-MIC evolution | This study |
| MP20-11 | MP06-01, mutant isolated during MTX sub-MIC evolution | This study |
| MP20-12 | MP06-01, mutant isolated during MTX sub-MIC evolution | This study |
| MP20-13 | MP06-01, mutant isolated during MTX sub-MIC evolution | This study |
| MP20-14 | MP06-01, mutant isolated during MTX sub-MIC evolution | This study |
| MP20-15 | MP06-01, mutant isolated during MTX sub-MIC evolution | This study |
| MP20-16 | MP06-01, mutant isolated during MTX sub-MIC evolution | This study |
| MP20-17 | MP06-01, mutant isolated during MTX sub-MIC evolution | This study |
| MP20-18 | MP06-01, mutant isolated during MTX sub-MIC evolution | This study |
| MP20-19 | MP06-01, mutant isolated during MTX sub-MIC evolution | This study |
| MP20-20 | MP06-01, mutant isolated during MTX sub-MIC evolution | This study |
| MP20-21 | MP06-01, mutant isolated during MTX sub-MIC evolution | This study |
| MP20-22 | MP06-01, mutant isolated during MTX sub-MIC evolution | This study |
| MP20-23 | MP06-01, mutant isolated during MTX sub-MIC evolution | This study |
| MP20-24 | MP06-01, mutant isolated during MTX sub-MIC evolution | This study |
| MP20-25 | MP06-01, mutant isolated during MTX sub-MIC evolution | This study |
| MP20-26 | MP06-01, mutant isolated during MTX sub-MIC evolution | This study |
| MP20-27 | MP06-01, mutant isolated during MTX sub-MIC evolution | This study |
| MP20-28 | MP06-01, mutant isolated during MTX sub-MIC evolution | This study |
| MP20-29 | MP06-01, mutant isolated during MTX sub-MIC evolution | This study |
| MP20-30 | MP06-01, mutant isolated during MTX sub-MIC evolution | This study |
| MP20-31 | MP06-01, mutant isolated during MTX sub-MIC evolution | This study |
| MP20-32 | MP06-01, mutant isolated during MTX sub-MIC evolution | This study |
| MP20-33 | MP06-01, mutant isolated during MTX sub-MIC evolution | This study |
| MP20-34 | MP06-01, mutant isolated during MTX sub-MIC evolution | This study |
| MP20-35 | MP06-01, mutant isolated during MTX sub-MIC evolution | This study |
| MP20-36 | MP06-01, mutant isolated during MTX sub-MIC evolution | This study |
| MP20-37 | MP06-01, mutant isolated during MTX sub-MIC evolution | This study |
| MP20-38 | MP06-01, mutant isolated during MTX sub-MIC evolution | This study |
| MP20-39 | MP06-01, mutant isolated during MTX sub-MIC evolution | This study |
| MP20-40 | MP06-01, mutant isolated during MTX sub-MIC evolution | This study |
| MP20-41 | MP06-01, mutant isolated during MTX sub-MIC evolution | This study |
| MP20-42 | MP06-01, mutant isolated during MTX sub-MIC evolution | This study |
| MP26-21 | *E. coli* W3110 Δ7NR*tolC* | (36) |
| MP26-52 | MP08-03 pG06-VIM-1 | This study |

^1^DA strain collection refers to the bacterial strain collection in the Dan Andersson lab at Uppsala University.

**Table S2:** Primer sequences used for strain constructions. Underline indicates the first 20 bp of the fluorescent protein gene whereas bold indicates the homology region to the *cat-sacB* cassette.

| **Primer name** | **5’ to 3’** | **bp** |
| --- | --- | --- |
| IS150_PCP25_cs_F | ATAGTACTGTTTAACTTTTCGAGACCTTAGGAGGTAAAAA**CATATGAATATCCTCCTTAGTTCC** | 64 |
| IS150_PCP25_cs_R | TAACCCTTAGTGACTCCTGCAGCGGCCGCTACTAGTATTA **TGTAGGCTGGAGCTGCTTC** | 59 |
| IS150_mtagbfp_F | ATAGTACTGTTTAACTTTTCGAGACCTTAGGAGGTAAAAA ATGAGCGAACTGATCAAAGAGA | 80 |
| IS150_syfp2_F | ATAGTACTGTTTAACTTTTCGAGACCTTAGGAGGTAAAAA ATGGTTAGCAAGGGCGAAGA | 80 |
| IS150_R | CCGAAGACTCTTACTCTTTCAATTTGCAGGCTAAAAACGC ATTACCGCCTTTGAGTGAGC | 80 |
| IS150_screen_F | TAAGATCCCTGCCATTTGG | 19 |
| IS150_screen_R | ATCAAGGAGAAGAAACAAACTT | 22 |
| dfrA1_fw | TTGTTAACCCTTTTGCCAGATTTGG | 25 |
| dfrA1_rv | CCTCTGAGGAAGAATTGTGAAACTATCACTAATGG | 35 |
| dfrA12_fw | CTAAAACAAAGTTATGCCATATGAACTCG | 29 |
| dfrA12_rv | CCGTTGACGGAATGGTTAGC | 20 |

**Table S3:** List of bioinformatic programs, including software versions, used for WGS analysis.

| **Tool** | **Version** |
| --- | --- |
| Abricate | 0.8.10 |
| BWA MEM | 0.7.17-r1188 |
| FastTree | 2.1.10 Double precision (No SSE3): |
| FreeBayes | 1.2.0-dirty |
| IQtree | 1.6.9 for Linux 64-bit built Dec 19 2018 |
| Kraken | 1.0 |
| MLST | 2.16.1 |
| MegaHit | 1.2.9 |
| Newick-Utils | 1.6 |
| Nullarbor | 2.0.20181010 |
| Prokka | 1.13.7 |
| Roary | 3.12.0 |
| SAMtools | 1.9 |
| SKESA | 2.3.0 |
| SPAdes | 3.13.0 |
| Snippy | 4.3.6 and 4.6.0 |
| Trimmomatic | 0.38 |
| centrifuge | 1.0.4 |
| seqret | 6.6.0.0 |
| seqtk | 1.3-r106 |
| snp-dists | 0.6.3 |
| Unicycler | v0.4.8 |
| makeblastdb | 2.9.0+ |
| tblastn | 2.9.0+ |
| bowtie2-build | 2.3.5 |
| bowtie2 | 2.3.5 |
| samtools | 1.9 |
| java | 11.0.1-internal |
| pilon | 1.23 |
| fastp | 0.19.7 |
| fastqc | 0.11.9 |

**Table S4:** MTX and TMP susceptibility profiles of strains used and constructed for the study. Strains with known TMP resistance determinants have been highlighted as bold in the table. The final MIC concentration was set as the modal value from 3-5 replicates (Table S5).

| **Strain** | **MTX MIC [mg/mL]** | **TMP MIC [μg/mL]** |
| --- | --- | --- |
| DA4201/DA5438 | 16 | 0·125 |
| DA56507 | 16 | 0·125 |
| DA52848 | 8 | 0·25 |
| DA12755 | >32 | 0·75 |
| **MP05-31** | **>32** | **>64** |
| MP06-01^1, 2^ | 4 | 0·225 |
| **MP06-05** | **>32** | **≥28^2^** |
| MP06-06 | 32 | 0·563^2^ |
| MP06-11 | 4 | 0·250^2^ |
| MP06-16 | 16 | 0·250^2^ |
| MP06-21 | 4 | 0·375^2^ |
| MP06-26 | 8 | 0·172^2^ |
| MP06-31 | 32 | 0·208^2^ |
| MP06-36 | 4 | 0·250^2^ |
| MP06-41 | 4 | 0·167^2^ |
| MP06-46 | 32 | 0·500^2^ |
| **MP18-03** | **>32** | **4** |
| **MP18-04** | **>32** | **32** |
| **MP18-05** | **>32** | **>64** |
| **MP18-06** | **>32** | **8** |
| **MP18-07** | **>32** | **32** |
| **MP18-08** | **>32** | **>64** |
| MP18-09 | <2 | 0·0625 |
| MP18-10 | <2 | 0·0625 |
| **MP18-11^3^** | **<2** | 0·0625 |
| **MP18-12** | **>32** | **>64** |
| MP26-21 | <0·25 |  |
| **MP26-52** | **>32** | **>64** |

^1^MP06-01 used as internal standard on all MXT-plates, modal MIC based on 50 replicates.

^2^TMP MICs determined in (29).

^3^MP18-11 has been marked in bold as the strain expresses the known TMP^R^ determinant *dfrA*1. The strain did however not confer resistance to either MTX or TMP.

**Table S5:** Raw data from IC_90_ measurements, presented as modal values throughout the paper. The output values of replicates that met quality control standards were read out on a two-fold scale. Each biological replicate is represented by B and the replicate number.

| **Strain** | **MTX MIC [mg/mL]** | | | | | | **TMP MIC [μg/mL]** | | | | | |
| --- | --- | --- | --- | --- | --- | --- | --- | --- | --- | --- | --- | --- |
|  | **B1** | **B2** | **B3** | **B4** | **B5** | **Modal** | **B1** | **B2** | **B3** | **B4** | **B5** | **Modal** |
| DA4201 | 16 | 16 | 8 | 32 |  | 16 | 0·125 | 0·125 | 0·125 | 0·125 |  | 0·125 |
| DA56507 | 16 | 8 | 16 | 8 | 16 | 16 |  |  |  |  |  | 0·25^1^ |
| DA52848 | 16 | 16 | 8 | 8 | 8 | 8 | 0·25 | 0·125 | 0·125 | 0·125 |  | 0·125 |
| DA12755 | 32 | 32 | >32 | >32 | 32 | 32 | 1 | 0·75 | 0·75 |  |  | 0·75 |
| MP05-31 | >32 | >32 | >32 |  |  | >32 | >64 | >64 | >64 |  |  | >64 |
| MP06-01 |  |  |  |  |  | 4^2^ |  |  |  |  |  |  |
| MP06-05 | >32 | >32 | >32 |  |  | >32 |  |  |  |  |  |  |
| MP06-06 | 32 | 16 | 32 |  |  | 32 |  |  |  |  |  |  |
| MP06-11 | 4 | 4 | 4 |  |  | 4 |  |  |  |  |  |  |
| MP06-16 | 16 | 16 | 4 |  |  | 16 |  |  |  |  |  |  |
| MP06-21 | 4 | 4 | 4 |  |  | 4 |  |  |  |  |  |  |
| MP06-26 | 16 | 8 | 8 |  |  | 8 |  |  |  |  |  |  |
| MP06-31 | 16 | 32 | 32 |  |  | 32 |  |  |  |  |  |  |
| MP06-36 | 4 | 2 | 4 |  |  | 4 |  |  |  |  |  |  |
| MP06-41 | 8 | 4 | 4 |  |  | 4 |  |  |  |  |  |  |
| MP06-46 | 32 | 32 | 8 |  |  | 32 |  |  |  |  |  |  |
| MP18-03 | >32 | >32 | >32 | >32 |  | >32 | 4 | 4 | 8 |  |  | 4 |
| MP18-04 | >32 | >32 | >32 |  |  | >32 | 8 | 32 | 32 | 32 | 32 | 32 |
| MP18-05 | >32 | >32 | >32 | >32 |  | >32 | >64 | >64 | >64 | >64 | >64 | >64 |
| MP18-06 | >32 | >32 | >32 |  |  | >32 | 8 | 8 | 8 |  |  | 8 |
| MP18-07 | >32 | >32 | >32 |  |  | >32 | 32 | 32 | 32 |  |  | 32 |
| MP18-08 | >32 | >32 | >32 |  |  | >32 | >64 | >64 | >64 | >64 | >64 | >64 |
| MP18-09 | <2 | <2 | <2 |  |  | <2 | 0·0625 | 0·0625 | 0·0625 | 0·0625 |  | 0·0625 |
| MP18-10 | <2 | <2 | <2 |  |  | <2 | 0·0625 | 0·0625 | 0·125 |  |  | 0·0625 |
| MP18-11 | <2 | <2 | <2 |  |  | <2 | 0·0625 | 0·0625 | 0·0625 |  |  | 0·0625 |
| MP18-12 | >32 | >32 | >32 |  |  | >32 | >64 | >64 | >64 |  |  | >64 |
| MP18-13 | 8 | 8 | 8 |  |  | 8 | 0·0625 | >0·03125 | >0·03125 | 0·03125 |  | >0·03125 |
| MP18-14 | 32 | 32 | 32 |  |  | 32 | 0·25 | 0·125 | 0·125 |  |  | 0·125 |
| MP18-15 | 4 | 4 | 4 |  |  | 4 | 0·25 | 0·125 | 0·125 |  |  | 0·125 |
| MP18-16 | >32 | >32 | >32 |  |  | >32 | 0·25 | 0·125 | 0·125 |  |  | 0·125 |
| MP18-17 | >32 | >32 | >32 |  |  | >32 | >64 | >64 | >64 |  |  | >64 |
| MP18-18 | >32 | >32 | >32 |  |  | >32 | 2 | 2 | 2 |  |  | 2 |
| MP18-19 | >32 | >32 | >32 |  |  | >32 | 4 | 4 | 4 |  |  | 4 |
| MP18-20 | >32 | >32 | >32 |  |  | >32 | 4 | 4 | 4 |  |  | 4 |
| MP18-21 | >32 | >32 | >32 |  |  | >32 | 4 | 4 | 4 |  |  | 4 |
| MP18-22 | >32 | >32 | >32 |  |  | >32 | 4 | 4 | 4 |  |  | 4 |
| MP18-23 | >32 | >32 | >32 |  |  | >32 | 8 | 8 | 8 |  |  | 8 |
| MP18-24 | >32 | >32 | >32 |  |  | >32 | 4 | 4 | 4 |  |  | 4 |
| MP18-25 | >32 | >32 | >32 |  |  | >32 | 8 | 4 | 4 |  |  | 4 |
| MP18-26 | >32 | >32 | >32 |  |  | >32 | 4 | 4 | 4 |  |  | 4 |
| MP18-27 | >32 | >32 | >32 |  |  | >32 | 4 | 4 | 4 |  |  | 4 |
| MP18-28 | >32 | >32 | >32 |  |  | >32 | 4 | 4 | 4 |  |  | 4 |
| MP26-21 | <2 | <0·25 | <0·25 | <0·25 |  | <0·25 |  |  |  |  |  |  |
| MP26-52 | >32 | >32 | >32 |  |  | >32 | >64 | >64 | >64 |  |  | >64 |

^1^The MIC for DA56507 was based on the modal from the following nine replicates: 0·25 - 0·25 - 0·125 - 0·125- 0·0625 - 0·0625 - 0·25 - 0·25 - 0·25

^2^MP06-01 was used as an internal standard on all MXT-plates, the MIC was based on the modal from the following 50 replicates: 4 - 4 - 4 - 8 - 4 - 8 - 4 - 4 - 4 - 4 - 8 - 4 - 4 - 4 - 8 - 2 - 4 - 4 - 4 - 4 - 4 - 4 - 8 - 4 - 8 - 8 - 8 - 8 - 4 - 4 - 2 - 2 - 2 - 2 - 4 - 2 - 8 - 4 - 4 - 4 - 4 - 4 - 8 - 4 - 8 - 8 - 8 - 8 - 4 – 8

**Table S6:** (1) Raw data from the BioScreen C instrument used to calculate the relative growth rates of a TMP^R^ K56-2 (MP06-05) compared to it’s susceptible parents strain (MP06-01) as well as the (2) BAT2.1 output file, including information on the strain and condition in each well. (3) Raw data from the BioScreen C instrument used to calculate the relative growth rates of a TMP^R^ K56-75 harbouring the pG06-VIM-1 plasmid (MP05-31) compared to it’s susceptible parents strain (MP06-41) as well as the (2) BAT2.1 output file, including information on the strain and condition in each well.

**Table S7:** The optical density measured for MP08-03 at 600 nm (OD_600_) following 18 hour shaking incubation at various concentrations of MTX. Six biological replicates were grown in independent cultures.

| **MTX [mg/mL]** | **MP08-03** | | | | | | | |
| --- | --- | --- | --- | --- | --- | --- | --- | --- |
|  | **B1** | **B2** | **B3** | **B4** | **B5** | **B6** | **Mean** | **St. dv.** |
| **0** | 0·953 | 0·929 | 0·927 | 0·979 | 0·964 | 0·976 | 0·955 | 0·002 |
| **1·5** | 0·515 | 0·507 | 0·255 | 0·372 | 0·522 | 0·539 | 0·452 | 0·114 |
| **2** | 0·450 | 0·449 | 0·327 | 0·268 | 0·391 | 0·511 | 0·399 | 0·090 |
| **3** | 0·478 | 0·434 | 0·421 | 0·311 | 0·445 | 0·482 | 0·428 | 0·062 |
| **4** | 0·333 | 0·270 | 0·297 | 0·300 | 0·349 | 0·398 | 0·324 | 0·046 |
| **6** | 0·363 | 0·327 | 0·280 | 0·300 | 0·352 | 0·357 | 0·330 | 0·034 |
| **8** | 0·249 | 0·253 | 0·254 | 0·309 | 0·294 | 0·324 | 0·281 | 0·033 |
| **12** | 0·236 | 0·219 | 0·261 | 0·256 | 0·244 | 0·260 | 0·246 | 0·017 |
| **16** | 0·200 | 0·193 | 0·239 | 0·239 | 0·229 | 0·215 | 0·219 | 0·020 |
| **24** | 0·129 | 0·151 | 0·155 | 0·164 | 0·160 | 0·161 | 0·153 | 0·013 |
| **32** | 0·099 | 0·107 | 0·166 | 0·184 | 0·132 | 0·138 | 0·138 | 0·033 |

**Table S8:** The optical density measured for MP26-52 at 600 nm (OD_600_) following 18 hour shaking incubation at various concentrations of MTX. Six biological replicates were grown in independent cultures.

| **MTX [mg/mL]** | **MP26-52** | | | | | | | |
| --- | --- | --- | --- | --- | --- | --- | --- | --- |
|  | **B1** | **B2** | **B3** | **B4** | **B5** | **B6** | **Mean** | **St. dv.** |
| **0** | 0·940 | 0·933 | 0·655 | 0·686 | 0·940 | 0·938 | 0·848 | 0·138 |
| **1·5** | 0·942 | 0·943 | 0·902 | 0·867 | 0·980 | 0·999 | 0·939 | 0·049 |
| **2** | 0·839 | 0·784 | 0·871 | 0·879 | 0·948 | 0·990 | 0·885 | 0·074 |
| **3** | 0·925 | 0·820 | 0·900 | 0·808 | 0·965 | 0·993 | 0·902 | 0·075 |
| **4** | 0·858 | 0·661 | 0·782 | 0·865 | 0·917 | 0·967 | 0·842 | 0·108 |
| **6** | 0·912 | 0·826 | 0·894 | 0·722 | 0·954 | 0·963 | 0·878 | 0·091 |
| **8** | 0·805 | 0·753 | 0·795 | 0·828 | 0·929 | 0·944 | 0·842 | 0·077 |
| **12** | 0·855 | 0·587 | 0·779 | 0·788 | 0·902 | 0·903 | 0·802 | 0·118 |
| **16** | 0·806 | 0·764 | 0·742 | 0·784 | 0·818 | 0·791 | 0·784 | 0·028 |
| **24** | 0·693 | 0·665 | 0·674 | 0·651 | 0·725 | 0·771 | 0·697 | 0·044 |
| **32** | 0·360 | 0·470 | 0·397 | 0·468 | 0·553 | 0·581 | 0·472 | 0·086 |

**Table S9:** Calculated selection coefficients of each replicate during head-to-head competitions between the TMP^R^ *folA* mutant (MP18-04 and MP18-07) and isogenic TMP^S^ (DA56507 and DA52848) strain.

| **MTX concentration [μg/mL]** | **0** | **200** | **400** | **600** | **800** | **1,000** |
| --- | --- | --- | --- | --- | --- | --- |
| Selection coefficients  *bfp*R:*yfp*S  (MP18-07:DA56507) | -0·0135075 | -0·0044673 | -0·0020299 | 0·03931029 | 0·03190433 | 0·07611339 |
|  | -0·0255208 | -0·0067445 | 0·00602582 | 0·03781498 | 0·04498202 | 0·08812079 |
|  | -0·0388344 | -0·0005859 | 0·00986104 | 0·0355561 | 0·07659162 | 0·09771619 |
|  | -0·0320059 | -0·0023467 | 0·00807802 | 0·04331207 | 0·05821313 | 0·1132978 |
|  | -0·0364165 | -0·0057047 | 0·00550519 | 0·0347153 | 0·04307795 | 0·12888063 |
|  | -0·0255894 | -0·0079951 | 0·01038676 | 0·01334624 | 0·04880345 | 0·09785017 |
| Selection coefficients  *yfp*R:*bfp*S  (MP18-04:DA52848) | -0·0372175 | -0·0005588 | 0·02263352 | 0·08257795 | 0·19266 | 0·1671059 |
|  | -0·0210616 | 0·00438223 | 0·04124424 | 0·12591823 | 0·15959803 | 0·31567517 |
|  | -0·0316734 | 0·0055524 | 0·03384889 | 0·12513247 | 0·1767099 | 0·26984198 |
|  | -0·0315623 | 0·00256964 | 0·04051869 | 0·08922083 | 0·20218262 | 0·24558123 |
|  | -0·0317976 | 0·00157104 | 0·02518501 | 0·09396005 | 0·18303477 | 0·19802551 |
|  | -0·0356574 | 0·00039645 | 0·04996337 | 0·09123521 | 0·1754452 | 0·25601346 |
| Mean | -0·0300703 | -0·0011609 | 0·02093506 | 0·06767498 | 0·11610025 | 0·17118518 |
| St. dv. | 0·00711319 | 0·00419485 | 0·01636382 | 0·03649801 | 0·06693214 | 0·07954708 |
| N | 12 | 12 | 12 | 12 | 12 | 12 |

**Table S10:** Calculated selection coefficients of each replicate during head-to-head competitions between the TMP^R^ *E. coli* MG1655 harboring the pG06-VIM-1 plasmid (MP18-05 and MP18-08) and isogenic TMP^S^ strain (DA56507 and DA52848).

| **MTX concentration [μg/mL]** | **0** | **25** | **50** | **75** | **100** | **200** |
| --- | --- | --- | --- | --- | --- | --- |
| Selection coefficients  *bfp*R:*yfp*S  (MP18-08:DA56507) | 0·0013778 | -0·0010507 | 0·0055619 | 0·01118738 | 0·01059141 | 0·02713763 |
|  | 0·00063436 | 0·00289697 | 0·00504481 | 0·01138153 | 0·01172904 | 0·03061104 |
|  | 0·00058383 | 0·00169671 | 0·00429196 | 0·00686043 | 0·01459478 | 0·03452533 |
|  | 0·0014676 | 0·0016126 | 0·00519585 | 0·01026467 | 0·01410065 | 0·02326364 |
|  | -0·0007107 | -0·0004971 | 0·00718521 | 0·00893631 | 0·00907501 | 0·03498757 |
|  | 0·00111876 | 0·0019667 | 0·00499255 | 0·01192992 | 0·01541848 | 0·03323181 |
| Selection coefficients  *yfp*R:*bfp*S  (MP18-05:DA52848) | 0·00370853 | 0·01542282 | 0·00627514 | 0·00868095 | 0·0212524 | 0·04832319 |
|  | 0·00665488 | 0·00014057 | 0·00940239 | 0·02035904 | 0·02432032 | 0·06098507 |
|  | 0·00594351 | 0·00041042 | 0·01254878 | 0·01541574 | 0·02045735 | 0·06160274 |
|  | 0·00515497 | 0·0072364 | 0·01024738 | 0·01097359 | 0·02139386 | 0·07722027 |
|  | 0·00331283 | 0·00814149 | 0·01039754 | 0·01232684 | 0·01975182 | 0·04729734 |
|  | 0·00567272 | 0·0017711 | 0·00961744 | 0·01822696 | 0·01961757 | 0·05169943 |
| Mean | 0·00290992 | 0·00331234 | 0·00756341 | 0·01221195 | 0·01685856 | 0·04424042 |
| St. dv. | 0·00238108 | 0·00453936 | 0·00262615 | 0·00378389 | 0·00470341 | 0·01573381 |
| N | 12 | 12 | 12 | 12 | 12 | 12 |

**Table S11:** Isogenic TMP^R^ and TMP^S^ *E. coli* MG1655 strains were competed against each other in six biological replicates, at different starting ratios and changes in *bfp*R:*yfp*S ratios over 30 generations were measured. Replicates where the starting ratio was not as intended and where random mutations have occurred during the competition experiment (reflected as a nonlinear slope) have been excluded from the calculations (34).

| **TMP^R^** | **MTX concentration** | **Replicate** | **Starting ratio** | **Ratio after**  **10 generations** | **Ratio after**  **20 generations** | **Ratio after**  **30 generations** |
| --- | --- | --- | --- | --- | --- | --- |
| *folA* | 400 μg/mL | 1 | 1 | 0·990 | 1·127 | 1·317 |
|  |  | 2 | 1 | 0·927 | 1·099 | 1·096 |
|  |  | 3 | 1 | 0·995 | 1·215 | 1·292 |
|  |  | 4 | 1 | 0·949 | 1·115 | 1·097 |
|  |  | 5 | 1 | 0·984 | 1·072 | 1·091 |
|  |  | 1 | 0·1 | 0·535 | 0·711 | 0·738 |
|  |  | 2 | 0·1 | 0·583 | 0·741 | 0·774 |
|  |  | 3 | 0·1 | 0·615 | 0·758 | 0·839 |
|  |  | 4 | 0·1 | 0·607 | 0·734 | 0·760 |
|  |  | 5 | 0·1 | 0·606 | 0·745 | 0·789 |
|  |  | 1 | 0·01 | 0·127 | 0·319 | 0·530 |
|  |  | 2 | 0·01 | 0·154 | 0·347 | 0·456 |
|  |  | 3 | 0·01 | 0·153 | 0·390 | 0·453 |
|  |  | 4 | 0·01 | 0·120 | 0·333 | 0·375 |
|  |  | 5 | 0·01 | 0·171 | 0·316 | 0·446 |
|  |  | 1 | 0·001 | 0·029 | 0·105 | 0·174 |
|  |  | 2 | 0·001 | 0·015 | 0·037 | 0·093 |
|  |  | 3 | 0·001 | 0·023 | 0·068 | 0·117 |
|  |  | 4 | 0·001 | 0·023 | 0·053 | 0·079 |
|  |  | 5 | 0·001 | 0·027 | 0·056 | 0·102 |
|  |  | 1 | 0·0001 | 0·002 | 0·004 | 0·006 |
|  |  | 2 | 0·0001 | 0·002 | 0·005 | 0·007 |
|  |  | 3 | 0·0001 | 0·003 | 0·008 | 0·015 |
|  |  | 5 | 0·0001 | 0·003 | 0·006 | 0·007 |
| pG06-VIM-1 | 75 μg/mL | 1 | 1 | 0·927 | 1·017 | 1·257 |
|  |  | 2 | 1 | 1·054 | 1·152 | 1·296 |
|  |  | 3 | 1 | 0·977 | 1·100 | 1·264 |
|  |  | 4 | 1 | 1·002 | 1·095 | 1·297 |
|  |  | 6 | 1 | 0·880 | 0·979 | 1·087 |
|  |  | 1 | 0·1 | 0·490 | 0·670 | 0·772 |
|  |  | 2 | 0·1 | 0·536 | 0·702 | 0·801 |
|  |  | 3 | 0·1 | 0·490 | 0·678 | 0·771 |
|  |  | 4 | 0·1 | 0·644 | 0·744 | 0·816 |
|  |  | 6 | 0·1 | 0·627 | 0·774 | 0·813 |
|  |  | 1 | 0·01 | 0·091 | 0·161 | 0·301 |
|  |  | 2 | 0·01 | 0·115 | 0·200 | 0·304 |
|  |  | 3 | 0·01 | 0·124 | 0·230 | 0·258 |
|  |  | 4 | 0·01 | 0·148 | 0·242 | 0·346 |
|  |  | 6 | 0·01 | 0·132 | 0·202 | 0·325 |
|  |  | 1 | 0·001 | 0·009 | 0·016 | 0·035 |
|  |  | 2 | 0·001 | 0·018 | 0·030 | 0·057 |
|  |  | 3 | 0·001 | 0·007 | 0·014 | 0·019 |
|  |  | 4 | 0·001 | 0·012 | 0·021 | 0·033 |
|  |  | 6 | 0·001 | 0·016 | 0·028 | 0·041 |
|  |  | 1 | 0·0001 | 0·001 | 0·001 | 0·004 |
|  |  | 2 | 0·0001 | 0·002 | 0·003 | 0·007 |
|  |  | 3 | 0·0001 | 0·002 | 0·004 | 0·004 |
|  |  | 5 | 0·0001 | 0·002 | 0·004 | 0·005 |
|  |  | 6 | 0·0001 | 0·001 | 0·002 | 0·005 |

**Table S12:** List of clones isolated during selective plating on high concentrations of MTX, including MTX and TMP susceptibility profiles as well as results from Sanger sequencing of the *folA* gene, its promotor region and of the *marR* gene. The final MIC concentration was set as the modal value from 3-4 replicates (Table S5). See Materials and Methods and Figure S3 for more detailed description on selection procedures.

| **Strain** | **[MTX] in**  **O.N. culture (mg/mL)** | **[MTX] in**  **plates (mg/mL)** | **MTX MIC (mg/mL)** | **TMP MIC (μg/mL)** | **Mutations in**  ***folA/*P*_folA_*** | **Mutations in *marR*** |
| --- | --- | --- | --- | --- | --- | --- |
| MP18-13 | 0 | 4 | 8 | <0·03125 | None | None |
| MP18-14 | 0 | 4 | 32 | 0·125 | None | None |
| MP18-15 | 0 | 8 | 4 | 0·125 | None | None |
| MP18-16 | 0 | 8 | >32 | 0·125 | None | None |
| MP18-17 | 0 | 16 | >32 | >64 | G>A 32 bp upstream | None |
| MP18-18 | 0 | 16 | >32 | 2 | G>A 32 bp upstream | None |
| MP18-19 | 0 | 16 | >32 | 4 | G>A 32 bp upstream | None |
| MP18-20 | 6·25 | 32 | >32 | 4 | C>T 58 bp upstream | A41E |
| MP18-21 | 6·25 | 32 | >32 | 4 | C>T 58 bp upstream | A41E |
| MP18-22 | 6·25 | 32 | >32 | 4 | C>T 58 bp upstream | A41E |
| MP18-23 | 6·25 | 32 | >32 | 8 | C>T 58 bp upstream | A41E |
| MP18-24 | 6·25 | 32 | >32 | 4 | C>T 58 bp upstream | A41E |
| MP18-25 | 6·25 | 32 | >32 | 4 | C>T 58 bp upstream | A41E |
| MP18-26 | 6·25 | 32 | >32 | 4 | C>T 58 bp upstream | A41E |
| MP18-27 | 6·25 | 32 | >32 | 4 | C>T 58 bp upstream | A41E |
| MP18-28 | 6·25 | 0 | >32 | 4 | C>T 58 bp upstream | A41E |

**Table S13:** Putative mutations identified during WGS analysis of MP18-13 when compared to the *E. coli* K56-2 WT strain used for selective plating. No mutations were found in genes known to play a role in antibiotic resistance.

| **Mutation**  **type** | **Original base** | **Alternative base** | **Evidence** | **Strand** | **Effect** | **Gene** | **Product** |
| --- | --- | --- | --- | --- | --- | --- | --- |
| SNP | G | T | T:605 G:0 | + | Asp577Tyr |  | gamma-glutamyltransferase |
| SNP | T | C | C:523 T:0 | + | Synonymous variant Phe411Phe | *proY* | proline-specific permease ProY |
| Deletion | CA | C | C:343 CA:0 |  |  |  |  |
| Insertion | A | AT | AT:405 A:0 |  |  |  |  |
| SNP | T | C | C:417 T:0 |  |  |  |  |
| SNP | A | C | C:634 A:0 | - | Cys594Gly |  | beta-galactosidase |
| SNP | G | T | T:610 G:0 | + | Asp241Tyr |  | 3-phenylpropionate MFS transporter |
| SNP | G | C | C:635 G:0 | - | Arg93Gly | *arnC* | undecaprenyl-phosphate 4-deoxy-4-formamido-L-arabinose transferase |
| SNP | G | C | C:629 G:0 | - | Leu91Val | *arnC* | undecaprenyl-phosphate 4-deoxy-4-formamido-L-arabinose transferase |
| SNP | C | T | T:635 C:0 | - | Synonymous variant Gly87Gly | *arnC* | undecaprenyl-phosphate 4-deoxy-4-formamido-L-arabinose transferase |
| SNP | G | C | C:713 G:0 | + | Synonymous variant Gly289Gly | *yfaL* | AIDA-I family autotransporter adhesin YfaL/EhaC |
| SNP | T | A | A:576 T:0 | - | Stop lost Ter116Tyrext*? | *yejF* | microcin C ABC transporter ATP-binding protein YejF |
| SNP | T | G | G:728 T:2 | + | Synonymous variant Pro229Pro |  | DEAD/DEAH box helicase family protein |
| SNP | A | G | G:712 A:52 | + | Met2Val |  | IS66 family transposase |
| Insertion | G | GA | GA:631 G:0 | + | Frameshift variant & stop lost Ter366fs |  | kfiB protein |
| SNP | G | C | C:552 G:0 | + | Synonymous variant Gly403Gly |  | purine permease |
| SNP | G | A | A:624 G:0 | + | Val115Ile | *nimT* | 2-nitroimidazole transporter |
| SNP | C | A | A:757 C:0 | + | Pro3Thr |  | biofilm-dependent modulation protein |
| SNP | C | A | A:609 C:0 | + | Ser557Tyr | *maeA* | oxaloacetate-decarboxylating malate dehydrogenase |
| Complex | CCCC | ACCT | ACCT:617 CCCC:0 | - | Synonymous variant |  | ABC transporter permease |
| SNP | C | T | T:678 C:0 | - | Val136Met | *tehB* | tellurite resistance methyltransferase TehB |
| SNP | G | A | A:287 G:23 | - | Synonymous variant Gly315Gly |  | EntS/YbdA MFS transporter |
| SNP | T | A | A:636 T:2 | + | Phe106Leu | *pdeR* | cyclic di-GMP phosphodiesterase |
| SNP | A | C | C:664 A:0 | + | Asn306His | *pdeR* | cyclic di-GMP phosphodiesterase |
| SNP | G | T | T:620 G:0 | - | Asp56Glu | *tonB* | TonB system transport protein TonB |
| SNP | T | C | C:603 T:0 | - | Synonymous variant Leu366Leu |  | autotransporter outer membrane beta-barrel domain-containing protein |
| Deletion | TC | T | T:630 TC:0 | - | Frameshift variant Asp86fs | *rfaP* | lipopolysaccharide core heptose(I) kinase RfaP |
| SNP | G | A | A:629 G:0 | - | Synonymous variant Asn433Asn | *ilvB* | acetolactate synthase large subunit |
| SNP | C | A | A:683 C:0 | - | Gly2778Val | *clbB* | colibactin hybrid non-ribosomal peptide synthetase/type I polyketide synthase ClbB |
| SNP | G | T | T:622 G:0 | + | Synonymous variant Gly538Gly | *ftsI* | peptidoglycan glycosyltransferase FtsI |

**Table S14:** Putative mutations identified during WGS analysis of MP18-17 when compared to the *E. coli* K56-2 WT strain used for selective plating. The strain was shown to have a G > A mutation 32 base pairs upstream of the *folA* gene, in its promotor, whereas no other mutations were found in genes known to play a role in antibiotic resistance.

| **Mutation type** | **Original base** | **Alternative base** | **Evidence** | **Strand** | **Effect** | **Gene** | **Product** |
| --- | --- | --- | --- | --- | --- | --- | --- |
| SNP | G | T | T:601 G:0 | + | Asp577Tyr |  | gamma-glutamyltransferase |
| SNP | T | C | C:490 T:0 | + | Synonymous variant Phe411Phe | *proY* | proline-specific permease ProY |
| Deletion | CA | C | C:360 CA:0 |  |  |  |  |
| Insertion | A | AT | AT:450 A:0 |  |  |  |  |
| SNP | T | C | C:384 T:0 |  |  |  |  |
| SNP | A | C | C:602 A:0 | - | Cys594Gly |  | beta-galactosidase |
| SNP | G | T | T:513 G:0 | + | Asp241Tyr |  | 3-phenylpropionate MFS transporter |
| SNP | G | C | C:635 G:0 | - | Arg93Gly | *arnC* | undecaprenyl-phosphate 4-deoxy-4-formamido-L-arabinose transferase |
| SNP | G | C | C:632 G:0 | - | Leu91Val | *arnC* | undecaprenyl-phosphate 4-deoxy-4-formamido-L-arabinose transferase |
| SNP | C | T | T:647 C:0 | - | Synonymous variant Gly87Gly | *arnC* | undecaprenyl-phosphate 4-deoxy-4-formamido-L-arabinose transferase |
| SNP | G | C | C:690 G:0 | + | Synonymous variant Gly289Gly | *yfaL* | AIDA-I family autotransporter adhesin YfaL/EhaC |
| SNP | T | A | A:547 T:0 | - | Stop lost Ter116Tyrext*? | *yejF* | microcin C ABC transporter ATP-binding protein YejF |
| SNP | A | C | C:735 A:0 | + | Asn75His |  | glycosyltransferase family 2 protein |
| SNP | T | G | G:604 T:0 | + | Synonymous variant Pro229Pro |  | DEAD/DEAH box helicase family protein |
| SNP | A | G | G:663 A:63 | + | Met2Val |  | IS66 family transposase |
| Insertion | G | GA | GA:558 G:1 | + | Frameshift variant & stop lost Ter366fs |  | kfiB protein |
| SNP | G | C | C:555 G:0 | + | Synonymous variant Gly403Gly |  | purine permease |
| SNP | G | A | A:572 G:0 | + | Val115Ile | *nimT* | 2-nitroimidazole transporter |
| SNP | C | A | A:800 C:0 | + | Pro3Thr |  | biofilm-dependent modulation protein |
| SNP | C | A | A:606 C:0 | + | Ser557Tyr | *maeA* | oxaloacetate-decarboxylating malate dehydrogenase |
| Complex | CCCC | ACCT | ACCT:525 CCCC:0 | - | Synonymous variant |  | ABC transporter permease |
| SNP | C | T | T:651 C:0 | - | Val136Met | *tehB* | tellurite resistance methyltransferase TehB |
| SNP | G | A | A:295 G:18 | - | Synonymous variant Gly315Gly |  | EntS/YbdA MFS transporter |
| SNP | T | A | A:622 T:2 | + | Phe106Leu | *pdeR* | cyclic di-GMP phosphodiesterase |
| SNP | A | C | C:564 A:0 | + | Asn306His | *pdeR* | cyclic di-GMP phosphodiesterase |
| SNP | G | T | T:496 G:0 | - | Asp56Glu | *tonB* | TonB system transport protein TonB |
| SNP | T | C | C:554 T:0 | - | Synonymous variant Leu366Leu |  | autotransporter outer membrane beta-barrel domain-containing protein |
| SNP | G | A | A:532 G:0 | - | Synonymous variant Asn433Asn | *ilvB* | acetolactate synthase large subunit |
| SNP | C | A | A:585 C:0 | - | Gly2778Val | *clbB* | colibactin hybrid non-ribosomal peptide synthetase/type I polyketide synthase ClbB |
| SNP | G | A | A:643 G:0 |  |  | Promotor region of *folA* | dihydrofolate reductase *folA* |
| SNP | G | T | T:638 G:0 | + | Synonymous variant Gly538Gly | *ftsI* | peptidoglycan glycosyltransferase FtsI |

**Table S15:** Putative mutations identified during WGS analysis of MP18-20 when compared to the *E. coli* K56-2 WT strain used for selective plating. The strain was shown to have a C>A mutation in the *marR* gene (A41G) and a C > T mutation 58 base pairs upstream of the *folA* gene, in its promotor. No other mutations were found in genes known to play a role in antibiotic resistance.

| **Mutation type** | **Original base** | **Alternative base** | **Evidence** | **Strand** | **Effect** | **Gene** | **Product** |
| --- | --- | --- | --- | --- | --- | --- | --- |
| SNP | G | T | T:643 G:0 | + | Asp577Tyr |  | gamma-glutamyltransferase |
| SNP | T | C | C:613 T:1 | + | Synonymous variant Phe411Phe | *proY* | proline-specific permease ProY |
| Deletion | CA | C | C:342 CA:0 |  |  |  |  |
| Insertion | A | AT | AT:398 A:1 |  |  |  |  |
| SNP | T | C | C:446 T:0 |  |  |  |  |
| SNP | A | C | C:636 A:0 | - | Cys594Gly |  | beta-galactosidase |
| SNP | G | T | T:579 G:0 | + | Asp241Tyr |  | 3-phenylpropionate MFS transporter |
| SNP | G | C | C:638 G:0 | - | Arg93Gly | *arnC* | undecaprenyl-phosphate 4-deoxy-4-formamido-L-arabinose transferase |
| SNP | G | C | C:640 G:0 | - | Leu91Val | *arnC* | undecaprenyl-phosphate 4-deoxy-4-formamido-L-arabinose transferase |
| SNP | C | T | T:645 C:0 | - | Synonymous variant Gly87Gly | *arnC* | undecaprenyl-phosphate 4-deoxy-4-formamido-L-arabinose transferase |
| SNP | G | C | C:740 G:0 | + | Synonymous variant Gly289Gly | *yfaL* | AIDA-I family autotransporter adhesin YfaL/EhaC |
| SNP | T | A | A:606 T:0 | - | Stop lost Ter116Tyrext*? | *yejF* | microcin C ABC transporter ATP-binding protein YejF |
| SNP | T | G | G:652 T:0 | + | Synonymous variant Pro229Pro |  | DEAD/DEAH box helicase family protein |
| SNP | A | C | C:680 A:0 | - | Phe148Cys |  | phosphoethanolamine transferase |
| SNP | A | G | G:726 A:66 | + | Met2Val |  | IS66 family transposase |
| Insertion | G | GA | GA:686 G:0 | + | Frameshift variant & stop lost Ter366fs |  | kfiB protein |
| SNP | G | C | C:598 G:0 | + | Synonymous variant Gly403Gly |  | purine permease |
| SNP | C | A | A:736 C:0 | + | Ala41Glu | *marR* | multiple antibiotic resistance transcriptional regulator MarR |
| SNP | G | A | A:580 G:0 | + | Val115Ile | *nimT* | 2-nitroimidazole transporter |
| SNP | C | A | A:895 C:0 | + | Pro3Thr |  | biofilm-dependent modulation protein |
| SNP | C | A | A:710 C:0 | + | Ser557Tyr | *maeA* | oxaloacetate-decarboxylating malate dehydrogenase |
| Complex | CCCC | ACCT | ACCT:585 CCCC:0 | - | Synonymous variant |  | ABC transporter permease |
| SNP | C | T | T:787 C:0 | - | Val136Met | *tehB* | tellurite resistance methyltransferase TehB |
| SNP | G | A | A:331 G:15 | - | Synonymous variant Gly315Gly |  | EntS/YbdA MFS transporter |
| SNP | T | A | A:674 T:0 | + | Phe106Leu | *pdeR* | cyclic di-GMP phosphodiesterase |
| SNP | A | C | C:630 A:0 | + | Asn306His | *pdeR* | cyclic di-GMP phosphodiesterase |
| SNP | G | T | T:595 G:0 | - | Asp56Glu | *tonB* | TonB system transport protein TonB |
| SNP | T | C | C:564 T:0 | - | Synonymous variant Leu366Leu |  | autotransporter outer membrane beta-barrel domain-containing protein |
| SNP | C | A | A:666 C:0 | - | Gly2778Val | *clbB* | colibactin hybrid non-ribosomal peptide synthetase/type I polyketide synthase ClbB |
| SNP | C | T | T:697 C:0 |  |  | Promotor region of *folA* | dihydrofolate reductase *folA* |
| SNP | G | T | T:626 G:0 | + | Synonymous variant Gly538Gly | *ftsI* | peptidoglycan glycosyltransferase FtsI |

**Table S16:** Putative mutations identified during WGS analysis of MP18-26 when compared to the *E. coli* K56-2 WT strain used for selective plating. The strain was shown to have a C>A mutation in the *marR* gene (A41G) and a C > T mutation 58 base pairs upstream of the *folA* gene, in its promotor. No other mutations were found in genes known to play a role in antibiotic resistance.

| **Mutation type** | **Original base** | **Alternative base** | **Evidence** | **Strand** | **Effect** | **Gene** | **Product** |
| --- | --- | --- | --- | --- | --- | --- | --- |
| SNP | G | T | T:620 G:0 | + | Asp577Tyr |  | gamma-glutamyltransferase |
| SNP | T | C | C:527 T:0 | + | Synonymous variant Phe411Phe | *proY* | proline-specific permease ProY |
| Deletion | CA | C | C:318 CA:0 |  |  |  |  |
| Insertion | A | AT | AT:360 A:0 |  |  |  |  |
| SNP | T | C | C:337 T:0 |  |  |  |  |
| SNP | A | C | C:565 A:0 | - | Cys594Gly |  | beta-galactosidase |
| SNP | G | T | T:475 G:0 | + | Asp241Tyr |  | 3-phenylpropionate MFS transporter |
| SNP | G | C | C:580 G:0 | - | Arg93Gly | *arnC* | undecaprenyl-phosphate 4-deoxy-4-formamido-L-arabinose transferase |
| SNP | G | C | C:573 G:0 | - | Leu91Val | *arnC* | undecaprenyl-phosphate 4-deoxy-4-formamido-L-arabinose transferase |
| SNP | C | T | T:577 C:0 | - | Synonymous variant Gly87Gly | *arnC* | undecaprenyl-phosphate 4-deoxy-4-formamido-L-arabinose transferase |
| SNP | G | C | C:628 G:0 | + | Synonymous variant Gly289Gly | *yfaL* | AIDA-I family autotransporter adhesin YfaL/EhaC |
| SNP | T | A | A:526 T:1 | - | Stop lost Ter116Tyrext*? | *yejF* | microcin C ABC transporter ATP-binding protein YejF |
| SNP | T | G | G:572 T:0 | + | Synonymous variant Pro229Pro |  | DEAD/DEAH box helicase family protein |
| SNP | A | C | C:607 A:0 | - | Phe148Cys |  | phosphoethanolamine transferase |
| SNP | A | G | G:581 A:45 | + | Met2Val |  | IS66 family transposase |
| Insertion | G | GA | GA:542 G:1 | + | Frameshift variant & stop lost Ter366fs |  | kfiB protein |
| SNP | G | C | C:525 G:0 | + | Synonymous variant Gly403Gly |  | purine permease |
| SNP | C | A | A:649 C:0 | + | Ala41Glu | *marR* | multiple antibiotic resistance transcriptional regulator MarR |
| SNP | G | A | A:570 G:0 | + | Val115Ile | *nimT* | 2-nitroimidazole transporter |
| SNP | C | A | A:715 C:0 | + | Pro3Thr |  | biofilm-dependent modulation protein |
| SNP | C | A | A:561 C:0 | + | Ser557Tyr | *maeA* | oxaloacetate-decarboxylating malate dehydrogenase |
| Complex | CCCC | ACCT | ACCT:427 CCCC:0 | - | Synonymous variant |  | ABC transporter permease |
| SNP | C | T | T:655 C:0 | - | Val136Met | *tehB* | tellurite resistance methyltransferase TehB |
| SNP | T | A | A:576 T:1 | + | Phe106Leu | *pdeR* | cyclic di-GMP phosphodiesterase |
| SNP | A | C | C:588 A:0 | + | Asn306His | *pdeR* | cyclic di-GMP phosphodiesterase |
| SNP | G | T | T:597 G:0 | - | Asp56Glu | *tonB* | TonB system transport protein TonB |
| SNP | T | C | C:511 T:0 | - | Synonymous variant Leu366Leu |  | autotransporter outer membrane beta-barrel domain-containing protein |
| SNP | C | A | A:548 C:0 | - | Gly2778Val | *clbB* | colibactin hybrid non-ribosomal peptide synthetase/type I polyketide synthase ClbB |
| SNP | C | T | T:527 C:0 |  |  | Promotor region of *folA* | dihydrofolate reductase *folA* |
| SNP | G | T | T:557 G:0 | + | Synonymous variant Gly538Gly | *ftsI* | peptidoglycan glycosyltransferase FtsI |

**Table S17:** Putative mutations identified during WGS analysis of MP18-28 when compared to the *E. coli* K56-2 WT strain used for selective plating. The strain was shown to have a C>A mutation in the *marR* gene (A41G) and a C > T mutation 58 base pairs upstream of the *folA* gene, in its promotor. No other mutations were found in genes known to play a role in antibiotic resistance.

| **Mutation type** | **Original base** | **Alternative base** | **Evidence** | **Strand** | **Effect** | **Gene** | **Product** |
| --- | --- | --- | --- | --- | --- | --- | --- |
| SNP | G | T | T:370 G:0 | + | Asp577Tyr |  | gamma-glutamyltransferase |
| SNP | T | C | C:371 T:0 | + | Synonymous variant Phe411Phe | *proY* | proline-specific permease ProY |
| Deletion | CA | C | C:208 CA:0 |  |  |  |  |
| ins | A | AT | AT:282 A:0 |  |  |  |  |
| SNP | T | C | C:238 T:0 |  |  |  |  |
| SNP | A | C | C:427 A:0 | - | Cys594Gly |  | beta-galactosidase |
| SNP | G | T | T:320 G:0 | + | Asp241Tyr |  | 3-phenylpropionate MFS transporter |
| SNP | G | C | C:370 G:0 | - | Arg93Gly | *arnC* | undecaprenyl-phosphate 4-deoxy-4-formamido-L-arabinose transferase |
| SNP | G | C | C:366 G:0 | - | Leu91Val | *arnC* | undecaprenyl-phosphate 4-deoxy-4-formamido-L-arabinose transferase |
| SNP | C | T | T:358 C:0 | - | Synonymous variant Gly87Gly | *arnC* | undecaprenyl-phosphate 4-deoxy-4-formamido-L-arabinose transferase |
| SNP | G | C | C:431 G:0 | + | Synonymous variant Gly289Gly | *yfaL* | AIDA-I family autotransporter adhesin YfaL/EhaC |
| SNP | T | A | A:332 T:0 | - | Stop lost Ter116Tyrext*? | *yejF* | microcin C ABC transporter ATP-binding protein YejF |
| SNP | T | G | G:399 T:0 | + | Synonymous variant Pro229Pro |  | DEAD/DEAH box helicase family protein |
| SNP | A | C | C:423 A:0 | - | Phe148Cys |  | phosphoethanolamine transferase |
| ins | G | GA | GA:366 G:0 | + | Frameshift variant & stop lost Ter366fs |  | kfiB protein |
| SNP | G | C | C:402 G:0 | + | Synonymous variant Gly403Gly |  | purine permease |
| SNP | C | A | A:419 C:0 | + | Ala41Glu | *marR* | multiple antibiotic resistance transcriptional regulator MarR |
| SNP | G | A | A:389 G:0 | + | Val115Ile | *nimT* | 2-nitroimidazole transporter |
| SNP | C | A | A:445 C:0 | + | Pro3Thr |  | biofilm-dependent modulation protein |
| SNP | C | A | A:402 C:0 | + | Ser557Tyr | *maeA* | oxaloacetate-decarboxylating malate dehydrogenase |
| Complex | CCCC | ACCT | ACCT:381 CCCC:0 | - | Synonymous variant |  | ABC transporter permease |
| SNP | C | T | T:382 C:0 | - | Val136Met | *tehB* | tellurite resistance methyltransferase TehB |
| SNP | G | A | A:210 G:10 | - | Gly315Gly |  | EntS/YbdA MFS transporter |
| SNP | T | A | A:396 T:0 | + | Phe106Leu | *pdeR* | cyclic di-GMP phosphodiesterase |
| SNP | A | C | C:402 A:0 | + | Asn306His | *pdeR* | cyclic di-GMP phosphodiesterase |
| SNP | G | T | T:368 G:0 | - | Asp56Glu | *tonB* | TonB system transport protein TonB |
| SNP | T | C | C:377 T:0 | - | Synonymous variant Leu366Leu |  | autotransporter outer membrane beta-barrel domain-containing protein |
| SNP | C | A | A:409 C:0 | - | Gly2778Val | *clbB* | colibactin hybrid non-ribosomal peptide synthetase/type I polyketide synthase ClbB |
| SNP | C | T | T:421 C:0 |  |  | Promotor region of *folA* | dihydrofolate reductase *folA* |
| SNP | G | T | T:422 G:0 | + | Synonymous variant Gly538Gly | *ftsI* | peptidoglycan glycosyltransferase FtsI |

**Table S18:** List of clones isolated on plates containing TMP 4 μg/mL during laboratory evolution of K56-2 (MP06-01) at 400 μg/mL MTX, including TMP MICs. The final MIC concentration was set as the modal value from 2-4 replicates. All isolates were found to have a single mutation (C>T) 58 bp upstream of the *folA* gene, in the *folA* promotor. Each biological replicate is represented by B and the replicate number.

| **Strain** | **Isolated from** | |  | **TMP MIC [μg/mL]** | | | |
| --- | --- | --- | --- | --- | --- | --- | --- |
|  | **Generation** | **Lineage** | **B1** | **B2** | **B3** | **B4** | **Modal** |
| MP19-78 | 50 | 1 | 8 | 12 | 12 |  | 12 |
| MP19-79 | 50 | 1 | 12 | 8 | 12 |  | 12 |
| MP19-80 | 50 | 1 | 8 | 16 | 16 |  | 16 |
| MP19-81 | 50 | 1 | 8 | 12 | 12 |  | 12 |
| MP20-01 | 50 | 1 | 12 | 12 |  |  | 12 |
| MP20-02 | 50 | 6 | 8 | 12 | 12 |  | 12 |
| MP20-03 | 50 | 6 | 12 | 12 |  |  | 12 |
| MP20-04 | 50 | 9 | 12 | 12 |  |  | 12 |
| MP20-05 | 50 | 9 | 8 | 12 | 8 |  | 8 |
| MP20-06 | 50 | 9 | 6 | 12 | 12 |  | 12 |
| MP20-07 | 50 | 9 | 8 | 8 |  |  | 8 |
| MP20-08 | 50 | 9 | 6 | 12 | 12 |  | 12 |
| MP20-09 | 100 | 1 | 12 | 12 |  |  | 12 |
| MP20-10 | 100 | 1 | 8 | 12 | 12 |  | 12 |
| MP20-11 | 100 | 1 | 8 | 8 |  |  | 8 |
| MP20-12 | 100 | 1 | 12 | 4 | 12 |  | 12 |
| MP20-13 | 100 | 1 | 12 | 12 |  |  | 12 |
| MP20-14 | 100 | 9 | 8 | 12 | 12 |  | 12 |
| MP20-15 | 100 | 9 | 12 | 12 |  |  | 12 |
| MP20-16 | 100 | 9 | 12 | 12 |  |  | 12 |
| MP20-17 | 100 | 9 | 12 | 8 | 12 |  | 12 |
| MP20-18 | 100 | 9 | 8 | 8 |  |  | 8 |
| MP20-19 | 150 | 1 | 8 | 8 |  |  | 8 |
| MP20-20 | 150 | 1 | 8 | 12 | 16 | 12 | 12 |
| MP20-21 | 150 | 1 | 8 | 8 |  |  | 8 |
| MP20-22 | 150 | 1 | 8 | 8 |  |  | 8 |
| MP20-23 | 150 | 1 | 8 | 8 |  |  | 8 |
| MP20-24 | 150 | 9 | 8 | 8 |  |  | 8 |
| MP20-25 | 150 | 9 | 12 | 12 |  |  | 12 |
| MP20-26 | 150 | 9 | 8 | 8 |  |  | 8 |
| MP20-27 | 150 | 9 | 8 | 12 | 12 |  | 12 |
| MP20-28 | 150 | 9 | 12 | 8 | 16 | 8 | 8 |
| MP20-29 | 200 | 9 | 8 | 8 |  |  | 8 |
| MP20-30 | 200 | 9 | 8 | 12 | 12 |  | 12 |
| MP20-31 | 200 | 9 | 6 | 8 | 12 | 8 | 8 |
| MP20-32 | 200 | 9 | 8 | 6 | 12 | 12 | 12 |
| MP20-33 | 200 | 9 | 12 | 12 |  |  | 12 |
| MP20-34 | 200 | 10 | 8 | 8 |  |  | 8 |
| MP20-35 | 250 | 9 | 8 | 8 |  |  | 8 |
| MP20-36 | 250 | 9 | 12 | 8 | 8 |  | 8 |
| MP20-37 | 250 | 9 | 16 | 12 | 12 |  | 12 |
| MP20-38 | 250 | 9 | 16 | 12 | 8 | 8 | 8 |
| MP20-39 | 250 | 10 | 16 | 24 | 16 |  | 16 |
| MP20-40 | 250 | 10 | 8 | 8 |  |  | 8 |
| MP20-41 | 250 | 10 | 12 | 12 |  |  | 12 |
| MP20-42 | 250 | 10 | 16 | 12 | 12 |  | 12 |
